# Association between violence against women and intimate partner characteristics: a population-based study conducted in Vitória, Espírito Santo^*^

**DOI:** 10.1590/1518-8345.7809.4967

**Published:** 2026-10-23

**Authors:** Franciéle Marabotti Costa Leite, Julya de Almeida Polverine, Dherik Fraga Santos, Bruna Venturin

**Affiliations:** 1 Universidade Federal do Espírito Santo, Vitória, ES, Brazil.; 2 Scholarship holder at the Conselho Nacional de Desenvolvimento Científico e Tecnológico (CNPq), Brazil.; 3 Universidade Federal do Espírito Santo, Departamento de Enfermagem, Vitória, ES, Brazil.; 4 Universidade Federal de Catalão, Departamento de Medicina, Catalão, GO, Brazil.; 5 Universidade Federal de Pelotas, Pelotas, RS, Brazil.

**Keywords:** Violence, Intimate Partner Violence, Domestic Violence, Violence Against Women, Risk Factors, Cross-Sectional Studies.

## Abstract

**Objective:**

to analyze the association between violence against women and the sociodemographic and behavioral characteristics of their intimate partners.

**Method:**

cross-sectional study conducted with 1,086 women aged 18 years or older who had an intimate partner in the past 24 months. The World Health Organization instrument on violence against women, validated for use in Brazil, was used to classify psychological, physical and sexual violence. The intimate partners’ sociodemographic and behavioral characteristics were collected. The results were presented as Prevalence Ratios and their respective 95% confidence intervals.

**Results:**

women whose partners were ≥41 years old had a 19% lower prevalence of psychological and physical violence. Higher prevalence of such abuse was found among women whose partners were black and who consumed alcohol. Psychological violence was 25%, 17%, and 30% higher, respectively, among those whose partners did not have a paid job, smoked or were former smokers. Physical violence was 73% more frequent among participants whose partners belonged to the first tertile of the *per capita* family income. Psychological, physical or sexual victimization was higher among those who felt fear of their partners (PR: 1.42; 95% CI: 1.24-1.64; PR: 2.52; 95% CI: 2.02-3.13; PR: 1.97; 95% CI: 1.48-2.63) and with controlling partners (PR: 1.48; 95% CI: 1.29-1.70; PR: 1.30; 95% CI: 1.04-1.62; PR: 1.43; 95% CI: 1.09-1.86).

**Conclusion:**

sociodemographic and behavioral characteristics of partners increase women’s vulnerability to violence.

## Highlights

**(1)**Higher prevalence of violence against women whose partners are controlling.

**(2)**Women who have ever felt afraid of their partners had a higher prevalence of violence.

**(3)**Alcohol use by the partner was associated with a higher frequency of violence.

**(4)**Condom refusal was associated with higher prevalence of violence.

**(5)**Encouraging gender equality policies and tackling forms of violence against women.

## Introduction

Violence against women is defined by the United Nations as any violent gender-based action that results in or is likely to result in physical, sexual or mental suffering for women^1^. Thus, intimate partner violence (IPV) is characterized by a common agent who acts in the violence and concomitantly relates to the victim, constituting an intimate partner. Furthermore, IPV is one of the most prevalent forms of violence against women and involves behaviors such as slapping, throwing objects that can hurt, pushing, and kicking^2^. Global estimates by the World Health Organization (WHO) indicate that 1 in 3 women experiences intimate partner violence in her lifetime, representing approximately 35% of women^1^. WHO also points out that 38% of murders against women were carried out by intimate partners^1^. 

Women around the world report suffering from violence perpetrated by their partners. According to a cross-sectional study carried out with female primary-care users in Vitória - ES (Brazil) who had an intimate partner, violence perpetrated by partners was associated with lack of occupation, refusal to use condoms during sex, controlling behavior, alcohol consumption, smoking habits and partners with less than 8 years of schooling^3^. In Wuhan, China, it was also observed that partners who smoked (33%) and consumed alcohol (35%) had a greater relationship with domestic violence than partners who did not smoke (23.89%) and did not drink (24.21%)^4^. In Europe, the prevalence of women who have suffered intimate partner violence at some point in their lives is 51.7%, and the study also agrees with the profile of intimate aggressors described: under 40 years of age, with no occupation or a lower salary than their partners^5^.

Violence against women holds short-, medium-, and long-term consequences for the mental and physical health of its victims^6^. With regard to the health of women in situations of physical and/or sexual violence by an intimate partner, according to WHO, there is a high rate of health problems, such as: they are more than twice as likely to have an abortion and experience depression, as well as 1.5 times more likely to contract HIV when compared to women who do not experience violence^1^. Exposure to violence can also lead to headaches, sexual disorders, phobias and prolonged fears^7^ ^-^ ^8^. 

According to a cross-sectional study, women who suffer with IPV are more likely to be late for their periodic cytopathological examination (pap smear), with a delay of 64% in victims of sexual violence, whereas in victims of physical violence the delay rate is twice as high^9^. As it has been shown, intimate partner violence continues to threaten the physical and mental health of women around the world. Despite the increase in surveys on the occurrence of intimate partner violence and its relationship with the demographic, socioeconomic and health profile of women, there is a scarcity of studies investigating the characteristics of intimate partners^10^ ^-^ ^11^. 

A systematic review on the profile of aggressors against women has already highlighted the importance of analyzing the characteristics of aggressors, providing information beyond sociodemographic data and addressing the behaviors and forms of psychological violence adopted by aggressors^10^ ^-^ ^11^. This is an important gap to be filled, and it can help the effectiveness of interventions regarding this problem. There is no population-based research on this topic conducted in the city where the study took place, and few population-based studies on the same topic in other locations in Brazil have been reported^12^. This investigation will contribute to a better understanding of the phenomenon.

It is worth noting that this study hypothesizes that partner behaviors, such as control and substance use, predict violence. In this context, this study aimed to analyze the association between violence against women and the sociodemographic and behavioral characteristics of their intimate partners.

## Method

### Type of study

This is a cross-sectional, analytical, population-based study based on the Strengthening the Reporting of Observational Studies in Epidemiology (STROBE) guidelines.

### Context

This study was conducted in the city of Vitória, Espírito Santo, Brazil. The city of Vitória, the capital of Espírito Santo state, was expected to hold a population of 369,534 people in 2021, in a territory of 97,123 km^2^, and a Municipal Human Development Index of 0.845. It has 79 neighborhoods and six Regional Health Areas^13^. According to data from the 2010 census, the female population represented 49.98% of the total population (163,853 women)^14^.

### Participants

The study population consisted of women aged 18 and over who had or had had an intimate partner in the last 24 months prior to the interview. In this study, an intimate partner was defined as a partner or a former partner and/or current boyfriend, as long as they were having sexual intercourse, regardless of whether they were in a formal marital relationship or not. 

### Selection criteria

As an exclusion criterion, women who were unable to understand or communicate due to intellectual or sensory deficit and who were, therefore, unable to respond to the research data collection instruments were excluded. 

### Sample definition

A multi-stage sampling process was adopted. The primary sampling unit was the census tracts of the city of Vitória provided by the 2010 Census, carried out by the Brazilian Institute of Geography and Statistics (IBGE).

To calculate the sample size, the estimated 50% prevalence of intimate partner violence in the study population was considered so as to maximize the sample, with a confidence level of 95% and an acceptable error of 5%. For studying associations between risk factors and outcomes, we specified a 95% confidence level and 80% power. Also, an exposed/unexposed ratio of 1:1 was considered. To this value, we added 10% for losses and 30% for confounding factors, thus reaching a necessary sample size of 1,100 women.

### Study variables

The independent variables relating to the sociodemographic characteristics of the women’s partners were age group in complete years; ethnicity/skin color; years of schooling; per capita family income in tertiles; and having a job. The behavioral variables analyzed were the use of illicit drugs; tobacco use; drinking alcohol; feeling afraid; being jealous; controlling; and refusing to use condoms.

The three types of violence against women (psychological, sexual, and physical) practiced by intimate partners throughout their lives were the dependent variables in this study. Each one was considered present when the woman answered “yes” to one of the items for each type of violence (psychological, physical or sexual).

### Instruments used to collect information

 An instrument containing the sociodemographic and behavioral characteristics was developed to obtain the independent variables. The ethnicity/skin color variable was self-reported according to IBGE: white, black and brown. 

To identify the outcomes, the short version of the original WHO questionnaire on violence against women was used. This questionnaire is validated for Brazil and aims to track the different forms of violence against women in their psychological, physical and sexual domains. This instrument has 13 questions and shows high internal consistency, as presented by Cronbach’s coefficients (an average of 0.88)^15^.

### Data collection

The pilot study took place in December 2021. Data were collected from January to May 2022 by a team of properly trained interviewers, all of whom were females. The research team also included field supervisors who were responsible for monitoring the field researchers regarding adherence to the data collection schedule, identifying and correcting flaws in the data collection process and/or inconsistencies in the data. The data collected in the pilot study did not comprise the final research sample. Field work began after the data obtained in the pilot study had been analyzed. The interviews were conducted at home, in a private place, respecting the participants’ privacy and confidentiality. All these measures were taken to minimize potential bias.

### Data processing and analysis

Descriptive analysis was carried out showing the relative frequencies. Heterogeneity and Fisher’s exact tests were used in the bivariate analysis according to assumptions. We assessed multicollinearity between the explanatory variables using the variance inflation factor, particularly due to the potential overlap among socioeconomic characteristics, and found no evidence of it, as observed by the Variance Inflation Factor (VIF) (>5 considered multicollinear). Poisson regression with robust variance was used to estimate the crude and adjusted prevalence ratios (PRs) and their respective 95% confidence intervals (95%CI). Initially, the variables associated with the outcomes under study (p<0.20) were included in the model in order to consider possible confounding factors. The backward selection method was used to exclude variables. In the final model, the 5% significance level (p<0.05) was adopted. Statistical analyses were carried out using the statistical program Stata^®^, version 15.1.

### Ethical aspects

The study was approved by the Research Ethics Committee of the Federal University of Espírito Santo, according to report no. 2.819.597. All the interviewees signed the Informed Consent Form and were informed of the objectives of the study and its possible risks and benefits.

## Results

As regards Table 1, it is noteworthy that most partners were aged 41 or over (63.3%); approximately 48% were white; almost 80% had 9 years or more of schooling; 34.0% were in the first family-income tertile, and 70.1% had an occupation. As to behavioral characteristics, approximately 6% used illicit drugs; 16.5% smoked, and almost 51% drank alcohol. According to the women, approximately 27% of their partners were jealous; almost 24% were controlling, and 10.4% reported feeling afraid of their male partners. Regarding the use of condoms, 16.9% had already refused to use them during sexual intercourse.

**Table 1:** Sociodemographic and behavioral characteristics of partners of women living in the city of Vitória, ES, Brazil, 2022 (N = 1,086)

Characteristics	%*	95%CI^†^
Sociodemographic		
Age group (N=1,085)		
Up to 40	36.7	33.9-39.6
41 years or more	63.3	60.4-66.1
**Ethnicity/Skin color (N=1,074)**		
White	47.5	44.5-50.5
Black	19.9	17.6-22.4
Brown	32.6	29.8-35.5
**Years of schooling (N=1,086)**		
Up to 8 years	20.3	18.0-22.8
9 years or more	79.7	77.2-82.0
***Per capita* family income (in tertiles) (N=1,067)**		
1^st^ tertile - the poorest	34.0	31.2-36.9
2^nd^ tertile	33.7	30.9-36.5
3^rd^ tertile - the richest	32.3	29.6-35.2
**Employed (N=1,079)**		
No	29.9	27.3-32.7
Yes	70.1	67.3-72.7
**Behavioral**		
**Uses illicit drugs (N=1,077)**		
No	94.1	92.4-95.3
Yes	5.9	4.7-7.5
**Uses tobacco (N=1,079)**		
No	76.2	73.5-78.6
Yes	16.5	14.4-18.8
Former smoker	7.3	5.9-9.0
**Drinks alcohol (N=1,086)**		
No	49.4	46.4-52.3
Yes	50.6	47.7-53.6
**Jealous (N=1,086)**		
No	73.3	70.6-75.8
Yes	26.7	24.2-29.4
**Already felt afraid of her partner (N=1,086)**		
No	89.6	87.6-91.3
Yes	10.4	8.7-12.4
**Controlling (N=1,086)**		
No	76.2	73.6-78.7
Yes	23.8	21.3-26.4
**Refuses condoms**		
No	83.1	80.8-85.3
Yes	16.9	14.7-19.2

*% = Relative frequency; **†**95%CI = 95% Confidence interval

It can be seen in Table 2 that there was an association between higher frequency of psychological, physical, and/or sexual violence throughout life and the following variables: partner’s ethnicity/skin color and partner’s years of schooling, *per capita* family income, drug use, smoking, jealous partners, controlling, feeling afraid of the partner and refusal of condoms during sexual intercourse (p<0.05). Furthermore, it was found that age and alcohol use were related to psychological and physical violence, and the variable employed was related to psychological violence.

**Table 2:** Prevalence of violence (psychological, physical and/or sexual) throughout life according to partners’ demographic, socioeconomic and behavioral characteristics. Vitória, ES, Brazil, 2022

	Psychological violence		Physical violence		Sexual violence	
Characteristics	P* (95% CI^†^)	P-value	P* (95% CI^†^)	P-value	P* (95% CI^†^)	P-value
Sociodemographic						
**Age group (N=1,085)**		<0.001^‡^		0.044^‡^		0.133^‡^
Up to 40	52.3 (47.3-57.1)		29.2 (24.9-33.8)		19.6 (16.0-23.8)	
41 years or more	41.2 (37.6-44.9)		23.6 (20.6-26.9)		15.9 (13.3-18.8)	
**Ethnicity/Skin color (N=1,074)**		0.001^§^		<0.001^§^		<0.001^§^
White	40.4 (36.2-44.7)		19.2 (16.0-22.9)		11.8 (9.2-14.9)	
Black	55.1 (48.4-61.7)		33.2 (27.2-39.8)		23.4 (18.2-29.5)	
Brown	46.6 (41.4-51.8)		30.6 (26.0-35.6)		21.4 (17.4-26.0)	
**Years of schooling (N=1,086)**		0.041^‡^		<0.001^‡^		0.005
Up to 8 years	51.4 (44.8-57.9)		36.4 (30.3-42.9)		23.6 (18.5-29.7)	
9 years or more	43.7 (40.4-47.0)		22.9 (20.2-25.8)		15.6 (13.3-18.2)	
***Per capita* family income (in tertiles) (N=1,067)**		<0.001		<0.001		0.001
1^st^ tertile - the poorest	53.4 (48.3-58.5)		37.5 (32.6-42.6)		23.1 (19.1-27.8)	
2^nd^ tertile	43.2 (38.1-48.4)		21.5 (17.5-26.0)		16.2 (12.7-20.3)	
3^rd^ tertile - the richest	39.1 (34.1-44.4)		18.3 (14.5-22.7)		12.8 (9.6-16.7)	
**Employed (N=1,079)**		0.006^§^		0.481^§^		0.517^§^
No	39.0 (33.8-44.4)		24.2 (19.8-29.1)		16.1 (12.5-20.5)	
Yes	48.0 (44.5-51.6)		26.2 (23.2-29.4)		17.7 (15.2-20.6)	
**Behavioral**						
**Uses illicit drugs (N=1,077)**		<0.001^‡^		<0.001^‡^		0.002^‡^
No	43.6 (40.6-46.7)		24.1 (21.5-26.8)		16.3 (14.1-18.7)	
Yes	70.3 (58.1-80.2)		46.9 (35.1-59.1)		31.3 (21.1-43.6)	
**Smokes (N=1,079)**		<0.001^§^		0.016^§^		0.004^§^
No	41.2 (37.9-44.6)		23.4 (20.6-26.4)		15.2 (12.9-17.8)	
Yes	60.7 (53.3-67.6)		32.6 (26.1-39.8)		24.2 (18.4-31.0)	
Former smoker	51.9 (41.0-62.7)		31.7 (22.4-42.7)		24.1 (15.9-34.7)	
**Drinks alcohol (N=1,086)**		<0.001^‡^		0.005^‡^		0.098^§^
No	38.6 (34.6-42.8)		21.8 (18.5-25.5)		15.3 (12.5-18.6)	
Yes	51.6 (47.5-55.8)		29.3 (25.6-33.2)		19.1 (16.0-22.6)	
**Jealous (N=1,086)**		<0.001^‡^		<0.001^‡^		<0.001^‡^
No	40.3 (37.0-43.8)		21.6 (18.9-24.6)		14.2 (11.9-16.8)	
Yes	58.6 (52.9-64.2)		36.6 (31.2-42.3)		25.5 (20.8-30.9)	
**Already felt afraid of her partner (N=1,086)**		<0.001^‡^		<0.001^‡^		<0.001^‡^
No	41.5 (38.5-44.7)		20.9 (18.4-23.5)		14.2 (12.1-16.5)	
Yes	77.0 (68.3-83.8)		66.4 (57.2-74.4)		43.4 (34.5-52.6)	
**Controlling (N=1,086)**		<0.001^‡^		<0.001^‡^		<0.001^‡^
No	38.7 (35.4-42.0)		21.0 (18.4-23.9)		13.7 (11.5-16.2)	
Yes	66.3 (60.3-71.8)		40.3 (34.5-46.4)		28.7 (23.5-34.5)	
**Refuses condoms**		<0.001^‡^		<0.001^‡^		<0.001^‡^
No	41.6 (38.5-44.9)		23.0 (20.4-25.9)		13.5 (11.4-15.9)	
Yes	62.8 (55.6-69.5)		38.3 (31.5-45.5)		35.5 (28.9-42.7)	

*P = Prevalence; 95% ^†^CI = 95% Confidence interval; ^‡^Fisher’s exact test; ^§^Chi-square test

After adjustments, Table 3 showed that women whose partners were older (aged 41 or over) experienced 19% lower prevalence of psychological violence. In addition, this type of abuse was more frequent among women with partners of black ethnicity/skin color (PR: 1.24; 95% CI: 1.06-1.44) and who did not have a job (PR: 1.25; 95% CI: 1.08-1.45). Partners who smoked (PR: 1.17; 95% CI: 1.01-1.36), former smokers (PR: 1.30; 95% CI: 1.04-1.61) and those who drank alcohol (PR: 1.19; 95% CI: 1.04-1.36) were representative of the characteristics showing a higher prevalence of psychological violence when compared to the reference group. Women who had felt afraid of their partners (PR: 1.42; 95% CI: 1.24-1.64), with controlling partners (PR: 1.48; 95% CI: 1.29-1.70) and with partners who refused to use condoms during sexual intercourse (PR: 1.24; 95% CI: 1.08-1.42) were among those who had experienced more psychological violence, conditioning for possible confounding factors (p<0.05).

**Table 3:** Crude and adjusted analysis of the lifetime prevalence of psychological violence according to partner characteristics. Vitória, ES, Brazil, 2022

Characteristics	Crude analysis		Adjusted analysis	
	PR* (95% CI^†^)	P-value	PR* (95% CI^†^)	P-value
**Age group**		<0.001		0.002
Up to 40 years	Ref.^‡^		Ref.^‡^	
41 years or more	0.79 (0.69-0.89)		0.81 (0.71-0.93)	
**Ethnicity/Skin color**		0.001		0.029
White	Ref.^‡^		Ref.^‡^	
Black	1.37 (1.16-1.60)		1.24 (1.06-1.44)	
Brown	1.15 (0.99-1.34)		1.09 (0.94-1.26)	
**Years of schooling**		0.033		0.697
Up to 8 years	1.18 (1.01-1.37)		0.97 (0.82-1.14)	
9 years or more	Ref.^‡^		Ref.^‡^	
***Per capita* family income (in tertiles)**		<0.001		0.232
1^st^ tertile - the poorest	1.37 (1.16-1.61)		1.15 (0.97-1.36)	
2^nd^ tertile	1.10 (0.92-1.32)		1.04 (0.88-1.23)	
3^rd^ tertile - the richest	Ref.^‡^		Ref.^‡^	
**Employed**		0.009		0.004
No	0.81 (0.70-0.95)		1.25 (1.08-1.45)	
Yes	Ref.^‡^		Ref.^‡^	
**Uses illicit drugs**		<0.001		0.854
No	Ref.^‡^		Ref.^‡^	
Yes	1.61 (1.35-1.92)		1.02 (0.82-1.27)	
**Smokes**		<0.001		0.016
No	Ref.^‡^		Ref.^‡^	
Yes	1.47 (1.27-1.70)		1.17 (1.01-1.36)	
Former smoker	1.26 (1.00-1.58)		1.30 (1.04-1.61)	
**Drinks alcohol**		<0.001		0.012
No	Ref.^‡^		Ref.^‡^	
Yes	1.34 (1.17-1.53)		1.19 (1.04-1.36)	
**Jealous**		<0.001		0.565
No	Ref.^‡^		Ref.^‡^	
Yes	1.45 (1.28-1.65)		1.04 (0.90-1.22)	
**Already felt afraid of her partner**		<0.001		<0.001
No	Ref.^‡^		Ref.^‡^	
Yes	1.85 (1.64-2.10)		1.42 (1.24-1.64)	
**Controlling**		<0.001		<0.001
No	Ref.^‡^		Ref.^‡^	
Yes	1.71 (1.52-1.94)		1.48 (1.29-1.70)	
**Refuses condoms**		<0.001		0.002
No	Ref.^‡^		Ref.^‡^	
Yes	1.51 (1.32-1.72)		1.24 (1.08-1.42)	

*PR = Prevalence ratio; 95% **†**CI = 95% Confidence interval; ^‡^Ref.= Reference group

In Table 4, the results of the adjusted analysis show that women with older partners (aged 41 or over) had a 19% lower lifetime prevalence of physical violence when compared to women with younger partners (p<0.05). Women with black partners experienced 33% more physical violence, and the prevalence of that condition was 73% higher among those with poorer partners, that is, those belonging to the first tertile of per capita family income. It was found that women with partners who consumed alcohol showed a prevalence of experiencing physical violence over their lifetime that was 1.27-fold that of women with partners who did not consume alcohol. It is noteworthy that the participants who reported feeling afraid of their partners showed higher prevalence of physical violence when compared to those who did not feel afraid, which was 2.52-fold higher The lifetime prevalence of physical violence was 30% higher among women with a controlling partner than among those without a controlling partner (p<0.05).

**Table 4:** Crude and adjusted analysis of lifetime prevalence of physical violence according to partner characteristics. Vitória, ES, Brazil, 2022

	Crude analysis		Adjusted analysis	
Characteristics	PR* (95% CI^†^)	P-value	PR* (95% CI^†^)	P-value
**Age group**		0.040		0.037
Up to 40 years	Ref.^‡^		Ref.^‡^	
41 years or more	0.81 (0.66-0.99)		0.81 (0.67-0.99)	
**Ethnicity/Skin color**		<0.001		0.085
White	Ref.^‡^		Ref.^‡^	
Black	1.73 (1.33-2.24)		1.33 (1.02-1.74)	
Brown	1.59 (1.25-2.02)		1.25 (0.98-1.59)	
**Years of schooling**		<0.001		0.590
Up to 8 years	1.59 (1.28-1.97)		1.06 (0.85-1.33)	
9 years or more	Ref.^‡^		Ref.^‡^	
***Per capita* family income (in tertiles)**		<0.001		<0.001
1^st^ tertile - the poorest	2.05 (1.58-2.66)		1.73 (1.34-2.24)	
2^nd^ tertile	1.17 (0.87-1.58)		1.12 (0.84-1.50)	
3^rd^ tertile - the richest	Ref.^‡^		Ref.^‡^	
**Employed**		0.484		
No	0.92 (0.73-1.16)		-	-
Yes	Ref.^‡^		-	-
**Uses illicit drugs**		<0.001		0.323
No	Ref.^‡^		Ref.^‡^	
Yes	1.95 (1.47-2.58)		1.18 (0.85-1.63)	
**Smokes**		0.013		0.072
No	Ref.^‡^		Ref.^‡^	
Yes	1.40 (1.09-1.78)		0.94 (0.74-1.21)	
Former smoker	1.35 (0.96-1.92)		1.43 (1.03-2.00)	
**Drinks alcohol**		0.005		0.017
No	Ref.^‡^		Ref.^‡^	
Yes	1.34 (1.09-1.65)		1.27 (1.04-1.54)	
**Jealous**		<0.001		0.620
No	Ref.^‡^		Ref.^‡^	
Yes	1.69 (1.38-2.07)		1.06 (0.84-1.33)	
**Already felt afraid of her partner**		<0.001		<0.001
No	Ref.^‡^		Ref.^‡^	
Yes	3.18 (2.66-3.81)		2.52 (2.02-3.13)	
**Controlling**		<0.001		0.019
No	Ref.^‡^		Ref.^‡^	
Yes	1.92 (1.57-2.34)		1.30 (1.04-1.62)	
**Refuses condoms**		<0.001		0.079
No	Ref.^‡^		Ref.^‡^	
Yes	1.66 (1.33-2.07)		1.22 (0.98-1.52)	

*PR = Prevalence ratio; 95% ^†^CI = 95% Confidence interval; ^‡^Ref. = Reference group

Table 5 shows that women with black and brown partners had 75% and 69% higher prevalence of sexual violence over their lifetime when compared to women with white partners. In addition, participants who reported feelings of fear towards their partners (PR: 1.97; 95% IC: 1.48-2.63) and who considered them controlling (PR: 1.43; 95% IC: 1.09-1.86) also showed higher prevalence of such abuse. Refusal to use a condom was associated with 2.18-fold more occurrences of sexual violence committed by an intimate partner (p < 0.05).

**Table 5:** Crude and adjusted analysis of lifetime prevalence of sexual violence according to partner characteristics. Vitória, ES, Brazil, 2022

	Crude analysis			Adjusted analysis	
Characteristics	PR* (95% CI^†^)	P-value	PR* (95% CI^†^)	P-value
**Age group**		0.116		0.190
Up to 40 years	Ref.^‡^		Ref.^‡^	
41 years or more	0.81 (0.62-1.05)		0.84 (0.64-1.09)	
**Ethnicity/Skin color**		<0.001		0.001
White	Ref.^‡^		Ref.^‡^	
Black	1.99 (1.41-2.79)		1.75 (1.26-2.43)	
Brown	1.82 (1.33-2.49)		1.69 (1.25-2.29)	
**Years of schooling**		0.004		0.750
Up to 8 years	1.52 (1.14-2.01)		1.05 (0.77-1.43)	
9 years or more	Ref.^‡^		Ref.^‡^	
***Per capita* family income (in tertiles)**		0.001		0.555
1^st^ tertile - the poorest	1.81 (1.30-2.53)		1.20 (0.85-1.72)	
2^nd^ tertile	1.27 (0.88-1.82)		1.08 (0.75-1.54)	
3^rd^ tertile - the richest	Ref.^‡^		Ref.^‡^	
**Employed**		0.519		
No	0.91 (0.68-1.22)		-	-
Yes	Ref.^‡^		-	-
**Uses illicit drugs**		0.001		0.421
No	Ref.^‡^		Ref.^‡^	
Yes	1.92 (1.30-2.83)		1.20 (0.77-1.86)	
**Smokes**		0.004		0.171
No	Ref.^‡^		Ref.^‡^	
Yes	1.59 (1.17-2.16)		1.18 (0.87-1.59)	
Former smoker	1.58 (1.04-2.42)		1.47 (0.95-2.25)	
**Drinks alcohol**		0.099		0.493
No	Ref.^‡^		Ref.^‡^	
Yes	1.25 (0.96-1.62)		1.10 (0.84-1.44)	
**Jealous**		<0.001		0.804
No	Ref.^‡^		Ref.^‡^	
Yes	1.80 (1.39-2.33)		1.04 (0.78-1.38)	
**Already felt afraid of her partner**		<0.001		<0.001
No	Ref.^‡^		Ref.^‡^	
Yes	3.06 (2.35-3.97)		1.97 (1.48-2.63)	
**Controlling**		<0.001		0.009
No	Ref.^‡^		Ref.^‡^	
Yes	2.10 (1.62-2.72)		1.43 (1.09-1.86)	
**Refuses condoms**		<0.001		<0.001
No	Ref.^‡^		Ref.^‡^	
Yes	2.63 (2.04-3.40)		2.18 (1.68-2.83)	

*PR = Prevalence ratio; ^†^95%CI = 95% Confidence interval; ^‡^Ref. = Reference group

## Discussion

The study showed an association between the sociodemographic and behavioral characteristics of intimate partners and the occurrence of violence against women throughout life. It was found that women with partners who had a controlling behavior, were jealous, refused to use condoms during sexual intercourse, were smokers or former smokers, used alcohol, did not have a job, belonged to the black ethnicity/skin color group and to the first tertile of *per capita* family income (the poorest) showed a higher prevalence of violence (psychological, physical, and/or sexual) throughout their lives. The findings are in line with national studies carried out among female primary care users^3^ and with puerperal women in a habitual risk maternity hospital^16^.

Women with younger partners, aged up to 40, had a higher prevalence of psychological and physical violence, after adjustments. The results of this study corroborate a national study carried out in 2003 with 251 female health service users in Porto Alegre, Rio Grande do Sul, which showed an association between the occurrence of psychological and physical violence and the partner’s age under 30^17^. An international study showed that women with older partners (aged 60 or over) were 43% less likely to suffer intimate partner violence than those with younger partners (18-29)^4^. 

The highest prevalence of physical violence was observed among women with partners belonging to the 1^st^ income tertile (the poorest) compared to those belonging to the richest tertile. It is crucial to contextualize that the association between greater poverty and greater recognition of violence against women should not be interpreted as a direct causal relationship, but as a reflection of structural conditions. Contexts of poverty often involve greater financial stress, precarious housing, less access to support networks and protection services, factors that can intensify conflicts and reduce alternatives for non-violent resolution. Furthermore, violence perpetrated by poor men tends to be more visible and less socially tolerated, while similar behaviors in higher socioeconomic groups may be normalized or concealed, affecting the recognition and reporting of violence^18^.

Other characteristics of the aggressor that were associated with higher probabilities of committing psychological, physical and sexual violence were black ethnicity/skin color. The socioeconomic and racial reality of Brazil shows that black Brazilian men have a lower level of education and occupy less prestigious positions when compared to white men^19^. Men and women with poorer education are more exposed to experiencing violence, with 91.8% of homicide victims being men, and 75.7% being black men^20^.

It is worth considering that this result may reflect racial bias in the perception of violent behaviors, where stereotypes associate black men with greater aggressiveness, leading to a faster or more intense recognition of violence when the aggressor is black than when he is white, even in similar situations - a phenomenon that has already been observed in studies on how race influences judgments of violence and culpability in intimate partner cases, for example, research on perceptions of intimate partner violence and jury decisions in racially differentiated scenarios indicates that the aggressor’s race alters how violence is evaluated^21^.

Furthermore, women with partners who did not work had a higher lifetime prevalence of psychological violence. Men without stable employment tend to exert greater coercive control over their partners, considering the context in which economic stress can be a significant predictor of violence^5^.

It was evidenced that women who self-reported fear of their partners had a higher prevalence of violence (psychological, physical and sexual) throughout their lives. Violent interpersonal relationships are often marked by fear and controlling behavior between partners^1^, so violent male behavior capable of justifying the fear suffered by these women is a relevant factor for physical and psychological consequences before and after the occurrence of violence. Such oppressive male behavior is related to the patriarchal culture and its structure in which men and women cannot be separated, thus reproducing the role of women’s subordination to men^22^.

The different spheres that can trigger fear in women in situations of domestic violence must be considered, and fear appears as an immobilizer and incapacitates reaction and breaking of the cycle^8^. As already discussed, fear can also be the result of a long series of threats from the aggressor as well as potentially damaging in the short and long term, increasing with the sense of impunity in which the State responds to violence^8^. It can also be based on threats to the safety of one’s own life and the lives of people close to them, such as children and family^22^.

Regarding condom use, the study found that women with partners who refused to use condoms had higher prevalence of psychological and sexual violence. In conjunction with the findings in this study, the literature reaffirms the evidence pointed out^3^ ^,^ ^16^ ^-^ ^17^. The result after adjustments found in this study for sexual violence was the same as that found in a study with female primary health care users in the same city^3^. The study showed that women with partners who refused to use condoms were nine times more likely to suffer sexual violence even during pregnancy^16^. The difficulty of negotiating both the use of condoms and other contraceptive methods points to the fragility of women’s decision-making power over their own sexual and reproductive health, evidencing a context in which this power belongs to the partner^3^ ^,^ ^16^ ^,^ ^23^.

In addition to the role of male domination when it comes to the decision to use condoms, there is also the domination of women’s sexual health. It is known that men who understand that condom use prevents diseases such as HIV also have a greater positive influence on their sexual relations with women, as opposed to men who did not understand sexual protection methods^24^. Additionally, a study carried out in Haiti showed that women’s refusal to have sexual intercourse was associated with physical violence and concomitant lower condom use rates^24^.

With regard to male controlling behavior, women who live in violent relationships may experience social distancing from family and friends as they live under constant surveillance and imposition of rules that violate human rights and harm their quality of life. In addition to social control, women have limited power in sexual circumstances, hindering the space for negotiating condom use and putting themselves at risk for violence^1^ ^,^ ^23^. 

In addition to gender inequality and inequality in power relations within the social and relationship structure, social inequality is also an important factor in not breaking the cycle of violence. Factors such as financial dependence^25^, low level of education^23^, worries about the future of children and emotional dependence are determining factors in women’s remaining in the cycle of violence^8^ ^,^ ^26^. Another relevant factor in the vulnerability of remaining in the context of violence is related to having experienced other types of domestic violence during one’s life, as well as a violent family context that can influence the naturalization of violence^27^. 

This study found a higher prevalence of psychological violence against women committed by men who were smokers or former smokers, a result also observed in a study conducted in the city of Shenzhen in China^28^. This is probably due to factors associated with smoking, such as stress, impulsivity and concomitant use of alcohol and other substances, which are already known as risk factors for violence^28^ ^-^ ^29^.

In this study, the use of alcohol by the intimate partner was associated with a higher prevalence of psychological and physical violence. Alcohol is a psychoactive substance, and its use affects neural connections and pathways. Also, depending on the amount consumed the effects can be increased, with possible consequences leading to the impairment of attention, cognition and dexterity; increased impulsivity; an aggressive profile; loss of behavioral control, and the exacerbation of reactions^30^. Therefore, the use of alcohol can increase the risk for violence due to the possibility of conflict and externalization of violent behavior, expressing an impulsive and aggressive side of partners who have a social construction with rooted *machismo* and patriarchy^3^ ^,^ ^5^ ^,^ ^16^.

In Brazil, men are the main consumers of excess alcohol when compared to women^30^. The attributable risk and hospital costs related to violence are high, and the total average cost of interpersonal violence from 2010 to 2018 due to alcohol can be estimated at more than 17 million dollars^26^. It is understood that the phenomenon under study is multidimensional. However, Brazil is one of the countries that has made slow progress in implementing public policies to tackle the harmful use of alcohol, with no intersectoral contact aimed at reducing the risk of violence^31^. 

The occurrence of violence throughout life can cause several physical, psychological, and social consequences in the short and long term for women with a history, and fear plus financial and emotional dependence can make it difficult to break the cycle of violence^1^. Health professionals are therefore a critical point of communication for these individuals^32^. However, in order to provide effective and efficient care for women in situations of violence, it is essential that health professionals develop their perspective on this serious public health problem, which encompasses different dimensions of human life (social, spiritual, physical, mental and biological). It is also necessary that this professional perspective have an interprofessional and intersectoral character so that the main referrals are made according to the real needs of the women being served^33^. In addition, it is important to highlight the compulsory notification that must be carried out by these professionals in cases or suspicions of violence. This has become a key tool for epidemiological surveillance and the formulation of public prevention and intervention policies^34^. 

The results of this study are of great relevance to clinical nursing and other health professionals, especially in the care of women in gynecological and obstetric consultations, as they reinforce the importance of knowing their partners’ profiles, considering that certain characteristics can lead to greater risk for violence. The findings, therefore, reinforce the need for a more complete and holistic data collection, as well as the need to report this problem and include women in the care and protection network.

It is the first population-based study to describe the profile of the current partner among women living in a capital city in the southeastern region of the country. Violence was measured using a validated instrument and women were guaranteed privacy to answer the entire questionnaire. It adds to the national and international literature on the profile of partners of women with a higher prevalence of violence throughout life, which has several gaps, so the information presented can support the planning and implementation of intersectoral public policies to combat violence against women.

Some limitations need to be mentioned, such as reverse causality, since this is a cross-sectional study and the temporality of the relationship is compromised, making it very important to conduct longitudinal studies. Social desirability bias can lead women who are victims of domestic violence to adjust their accounts to conform to social norms or the researcher’s expectations. This can result in minimizing the severity of violence by omitting episodes or describing the perpetrator in a less negative light, especially when there is fear of judgment or stigmatization. Furthermore, feelings of guilt, emotional or economic dependence can influence more defensive responses towards the perpetrator. Thus, this bias can compromise the accuracy and completeness of data on the violence suffered. For the reasons already mentioned, there may also be residual confounding.

## Conclusion

The results showed an association between violence against women and the socioeconomic and behavioral characteristics of their intimate partners. It is known that the phenomenon of violence is complex and multicausal, but this study demonstrates that characteristics, especially behavioral characteristics of intimate partners, have a stronger association with the occurrence of violence. These data present a path for evidence-based intervention measures, since behavior can be modified as long as efficient interventions are applied. The findings highlight the importance of intersectoral action to promote a culture of peace, address violence, social and gender inequality, as well as the need to reduce the use of alcohol, tobacco and other drugs.

## Data Availability

Datasets related to this article will be available upon request to the corresponding author.
