# Supplementary material for: Association between violence against women and intimate partner characteristics: a population-based study conducted in Vitória, Espírito Santo
Source: Rev Lat Am Enfermagem. 2026 Oct 23;34:e4967. doi: 10.1590/1518-8345.7809.4967 (PMC13706262; doi:10.1590/1518-8345.7809.4967)
Supplement: Translation

# Translation: Associação entre a violência contra as mulheres e as características do parceiro íntimo: um estudo de base populacional realizado em Vitória, Espírito Santo^*^

**DOI:** 10.1590/1518-8345.7809.4968

**Authors:** Franciéle Marabotti Costa Leite, Julya de Almeida Polverine, Dherik Fraga Santos, Bruna Venturin

**Affiliations:** 1 Universidade Federal do Espírito Santo, Vitória, ES, Brasil.; 2 Scholarship holder at the Conselho Nacional de Desenvolvimento Científico e Tecnológico (CNPq), Brasil.; 3 Universidade Federal do Espírito Santo, Departamento de Enfermagem, Vitória, ES, Brasil.; 4 Universidade Federal de Catalão, Departamento de Medicina, Catalão, GO, Brasil.; 5 Universidade Federal de Pelotas, Pelotas, RS, Brasil.

**Keywords:** Violência, Violência por Parceiro Íntimo, Violência Doméstica, Violência Contra a Mulher, Fatores de Risco, Estudos Transversais.

## Abstract

**Objetivo:**

analisar a associação entre a violência contra as mulheres e as características sociodemográficas e comportamentais de seus parceiros íntimos.

**Método:**

estudo transversal realizado com 1.086 mulheres com 18 anos ou mais que tiveram um parceiro íntimo nos últimos 24 meses. Utilizou-se o instrumento da Organização Mundial da Saúde sobre violência contra as mulheres, validado para uso no Brasil, para classificar a violência psicológica, física e sexual. Foram coletadas as características sociodemográficas e comportamentais dos parceiros íntimos. Os resultados foram apresentados como Razões de Prevalência e seus respectivos intervalos de confiança de 95%.

**Resultados:**

mulheres cujos parceiros tinham ≥41 anos apresentaram prevalência 19% menor de violência psicológica e física. Prevalências maiores desses abusos foram encontradas entre mulheres cujos parceiros eram pretos e consumiam álcool. A violência psicológica foi 25%, 17% e 30% maior, respectivamente, entre aquelas cujos parceiros não tinham emprego remunerado, fumavam ou eram ex-fumantes. A violência física foi 73% mais frequente entre as participantes cujos parceiros pertenciam ao primeiro tercil da renda familiar *per capita*. A vitimização psicológica, física ou sexual foi maior entre aquelas que sentiam medo de seus parceiros (RP: 1,42; IC 95%: 1,24-1,64; RP: 2,52; IC 95%: 2,02-3,13; RP: 1,97; IC 95%: 1,48-2,63) e com parceiros controladores (RP: 1,48; IC 95%: 1,29-1,70; RP: 1,30; IC 95%: 1,04-1,62; RP: 1,43; IC 95%: 1,09-1,86).

**Conclusão:**

as características sociodemográficas e comportamentais dos parceiros aumentam a vulnerabilidade das mulheres à violência.

## Destaques

**(1)** Maior prevalência de violência contra mulheres cujos parceiros são controladores.

**(2)** As mulheres que já sentiram medo de seus parceiros apresentaram maior prevalência de violência.

**(3)** O consumo de álcool pelo parceiro foi associado a uma maior frequência de violência.

**(4)** A recusa ao uso de preservativo foi associada a maior prevalência de violência.

**(5)** Incentivar políticas de igualdade de gênero e combater as formas de violência contra as mulheres.

## Introdução

A violência contra as mulheres é definida pelas Nações Unidas como qualquer ato violento de natureza de gênero que resulte ou possa resultar em sofrimento físico, sexual ou mental para as mulheres^1^. Assim, a violência cometida pelo parceiro íntimo (VPI) é caracterizada por um agente comum que pratica a violência e, ao mesmo tempo, mantém uma relação com a vítima, constituindo-se como parceiro íntimo. Além disso, a VPI é uma das formas mais prevalentes de violência contra as mulheres e envolve comportamentos como dar tapas, atirar objetos que possam causar ferimentos, empurrar e chutar^2^. Estimativas globais da Organização Mundial da Saúde (OMS) indicam que 1 em cada 3 mulheres sofre violência por parceiro íntimo ao longo da vida, o que representa aproximadamente 35% das mulheres^1^. A OMS também destaca que 38% dos homicídios contra mulheres foram cometidos por parceiros íntimos^1^.

Mulheres em todo o mundo relatam sofrer violência por parte de seus parceiros. De acordo com um estudo transversal realizado com mulheres usuárias de atenção primária em Vitória - ES (Brasil) que tinham um parceiro íntimo, a violência por parte dos parceiros foi associada à falta de ocupação, à recusa em usar preservativos durante as relações sexuais, ao comportamento controlador, ao consumo de álcool, ao tabagismo e a parceiros com menos de 8 anos de escolaridade^3^. Em Wuhan, na China, também foi observado que parceiros que fumavam (33%) e consumiam álcool (35%) apresentavam maior relação com a violência doméstica do que parceiros que não fumavam (23,89%) e não bebiam (24,21%)^4^. Na Europa, a prevalência de mulheres que sofreram violência por parceiros íntimos em algum momento de suas vidas é de 51,7%, e o estudo também confirma o perfil dos agressores íntimos descrito: com menos de 40 anos de idade, sem ocupação ou com salário inferior ao de suas parceiras^5^.

A violência contra as mulheres acarreta consequências a curto, médio e longo prazo para a saúde mental e física das vítimas^6^. No que diz respeito à saúde das mulheres em situações de violência física e/ou sexual por parte de um parceiro íntimo, de acordo com a OMS, há uma alta incidência de problemas de saúde, tais como: elas têm duas vezes mais chances de realizar um aborto e sofrer de depressão, bem como 1,5 vez mais chance de contrair o HIV quando comparadas a mulheres que não sofrem violência^1^. A exposição à violência também pode levar a dores de cabeça, distúrbios sexuais, fobias e medos prolongados^7^ ^-^ ^8^.

De acordo com um estudo transversal, as mulheres vítimas de violência por parceiro íntimo (VPI) apresentam maior probabilidade de atrasar o exame citopatológico periódico (Papanicolau), com um atraso de 64% entre as vítimas de violência sexual, enquanto entre as vítimas de violência física, a taxa de atraso é duas vezes maior^9^. Conforme demonstrado, a violência por parceiro íntimo continua a ameaçar a saúde física e mental das mulheres em todo o mundo. Apesar do aumento de pesquisas sobre a ocorrência de violência por parceiro íntimo e sua relação com o perfil demográfico, socioeconômico e de saúde das mulheres, há escassez de estudos que investiguem as características dos parceiros íntimos^10^ ^-^ ^11^. 

Uma revisão sistemática sobre o perfil dos agressores contra mulheres já destacou a importância de analisar as características dos agressores, fornecendo informações além dos dados sociodemográficos e abordando os comportamentos e as formas de violência psicológica adotadas pelos agressores^10^ ^-^ ^11^. Essa é uma lacuna importante a ser preenchida e pode contribuir para a eficácia das intervenções relacionadas a esse problema. Não há pesquisas de base populacional sobre esse tema realizadas na cidade onde o estudo foi realizado, e poucos estudos de base populacional sobre o mesmo tema em outras localidades do Brasil foram relatados^12^. Esta investigação contribuirá para uma melhor compreensão do fenômeno.

Vale ressaltar que este estudo parte da hipótese de que comportamentos do parceiro, como o controle e o uso de substâncias, são fatores preditivos da violência. Nesse contexto, o presente estudo teve como objetivo analisar a associação entre a violência contra as mulheres e as características sociodemográficas e comportamentais de seus parceiros íntimos.

## Método

### Tipo de estudo

Este é um estudo transversal, analítico e de base populacional, elaborado de acordo com as diretrizes do *Strengthening the Reporting of Observational Studies in Epidemiology* (STROBE).

### Contexto 

Este estudo foi realizado na cidade de Vitória, no Espírito Santo, Brasil. Estima-se que a cidade de Vitória, capital do estado do Espírito Santo, tenha uma população de 369.534 habitantes em 2021, em um território de 97.123 km^2^_,_ e um Índice de Desenvolvimento Humano Municipal de 0,845. Possui 79 bairros e seis Regionais de Saúde^13^. De acordo com dados do Censo de 2010, a população feminina representava 49,98% da população total (163.853 mulheres)^14^.

### Participantes

A população do estudo foi composta por mulheres com 18 anos ou mais que tinham ou haviam tido um parceiro íntimo nos últimos 24 meses anteriores à entrevista. Neste estudo, um parceiro íntimo foi definido como um parceiro ou ex-parceiro e/ou namorado atual, desde que mantivessem relações sexuais, independentemente de estarem ou não em uma relação conjugal formal.

### Critérios de seleção 

Como critério de exclusão, foram excluídas as mulheres que não conseguiam compreender ou comunicar-se devido a um déficit intelectual ou sensorial e que, por isso, não conseguiam responder aos instrumentos de coleta de dados da pesquisa.

### Definição da amostra

Foi adotado um processo de amostragem multietapas. A unidade primária de amostragem foram os setores censitários da cidade de Vitória, fornecidos pelo Censo de 2010, realizado pelo Instituto Brasileiro de Geografia e Estatística (IBGE).

Para calcular o tamanho da amostra, considerou-se a prevalência estimada de 50% da violência por parceiro íntimo na população do estudo, a fim de maximizar a amostra, com um nível de confiança de 95% e um erro aceitável de 5%. Para estudar as associações entre fatores de risco e desfechos, especificamos um nível de confiança de 95% e um poder de 80%. Além disso, foi considerada uma proporção de expostas/não expostas de 1:1. A esse valor, adicionamos 10% para perdas e 30% para fatores de confusão, chegando assim a um tamanho de amostra necessário de 1.100 mulheres.

### Variáveis de estudo

As variáveis independentes relacionadas às características sociodemográficas dos parceiros das mulheres foram: faixa etária em anos completos; etnia/cor da pele; anos de escolaridade; renda familiar *per capita* em tercis; e ter emprego. As variáveis comportamentais analisadas foram: uso de drogas ilícitas; tabagismo; consumo de álcool; sentir medo; sentir ciúmes; ser controlador; e recusar-se a usar preservativos.

Os três tipos de violência contra as mulheres (psicológica, sexual e física) praticados por parceiros íntimos ao longo de suas vidas constituíram as variáveis dependentes deste estudo. Considerou-se que cada um deles estava presente quando a mulher respondia “sim” a um dos itens relativos a cada tipo de violência (psicológica, física ou sexual).

### Instrumentos utilizados para a coleta de informações

Foi desenvolvido um questionário contendo características sociodemográficas e comportamentais para obter as variáveis independentes. A variável etnia/cor da pele foi autorrelatada de acordo com o IBGE: branca, preta e parda.

Para identificar os resultados, foi utilizada a versão resumida do questionário original da OMS sobre violência contra as mulheres. Esse questionário foi validado para o Brasil e tem como objetivo monitorar as diferentes formas de violência contra as mulheres nos domínios psicológico, físico e sexual. Esse instrumento conta com 13 perguntas e apresenta alta consistência interna, conforme demonstrado pelos coeficientes de Cronbach (uma média de 0,88)^15^.

### Coleta de dados

O estudo-piloto foi realizado em dezembro de 2021. Os dados foram coletados de janeiro a maio de 2022 por uma equipe de entrevistadoras devidamente treinadas. A equipe de pesquisa também contou com supervisoras de campo, responsáveis por monitorar as pesquisadoras de campo quanto ao cumprimento do cronograma de coleta de dados, bem como por identificar e corrigir falhas no processo de coleta e/ou inconsistências nos dados. Os dados coletados no estudo-piloto não compuseram a amostra final da pesquisa. O trabalho de campo teve início após a análise dos dados obtidos no estudo-piloto. As entrevistas foram realizadas em domicílio, em local privado, respeitando a privacidade e a confidencialidade dos participantes. Todas essas medidas foram tomadas para minimizar possíveis vieses.

### Processamento e análise dos dados

Foi realizada uma análise descritiva para apresentar as frequências relativas. Os testes de heterogeneidade e o teste exato de Fisher foram utilizados na análise bivariada, de acordo com os pressupostos. Avaliamos a multicolinearidade entre as variáveis explicativas utilizando o fator de inflação da variância, especialmente devido à possível sobreposição entre as características socioeconômicas, e não encontramos evidências disso, conforme observado pelo Fator de Inflação da Variância (VIF) (valor >5 é considerado indicativo de multicolinearidade). Utilizou-se a regressão de Poisson com variância robusta para estimar as razões de prevalência (RP) brutas e ajustadas e seus respectivos intervalos de confiança de 95% (IC 95%). Inicialmente, as variáveis associadas aos desfechos em estudo (p < 0,20) foram incluídas no modelo a fim de considerar possíveis fatores de confusão. O método de seleção regressiva foi utilizado para excluir variáveis. No modelo final, foi adotado o nível de significância de 5% (p < 0,05). As análises estatísticas foram realizadas utilizando o programa estatístico Stata^®^, versão 15.1.

### Aspectos éticos

O estudo foi aprovado pelo Comitê de Ética em Pesquisa da Universidade Federal do Espírito Santo, conforme o parecer n.º 2.819.597. Todos os entrevistados assinaram o Termo de Consentimento Livre e Esclarecido e foram informados sobre os objetivos do estudo e seus possíveis riscos e benefícios.

## Resultados

No que diz respeito à Tabela 1, vale destacar que a maioria dos parceiros tinha 41 anos ou mais (63,3%); aproximadamente 48% eram brancos; quase 80% tinham 9 anos ou mais de escolaridade; 34,0% estavam no primeiro tercil de renda familiar e 70,1% exerciam alguma atividade profissional. Quanto às características comportamentais, aproximadamente 6% usavam drogas ilícitas; 16,5% fumavam e quase 51% consumiam álcool. Segundo as mulheres, aproximadamente 27% de seus parceiros eram ciumentos; quase 24% eram controladores e 10,4% relataram sentir medo de seus parceiros. Em relação ao uso de preservativos, 16,9% já haviam se recusado a usá-los durante a relação sexual.

**Tabela 1:** Características sociodemográficas e comportamentais dos parceiros de mulheres residentes na cidade de Vitória, ES, Brasil, 2022 (N = 1.086)

Características	%*	IC 95%^†^
Sociodemográficas		
Faixa etária (N=1.085)		
Até 40 anos	36,7	33,9-39,6
41 anos ou mais	63,3	60,4-66,1
**Etnia/Cor da pele (N=1.074)**		
Branco	47,5	44,5-50,5
Preto	19,9	17,6-22,4
Pardo	32,6	29,8-35,5
**Escolaridade (N=1.086)**		
Até 8 anos	20,3	18,0-22,8
9 anos ou mais	79,7	77,2-82,0
**Renda familiar *per capita* (em tercis) (N=1.067)**		
1.º tercil - os mais pobres	34,0	31,2-36,9
2.º tercil	33,7	30,9-36,5
3.º tercil - os mais ricos	32,3	29,6-35,2
**Empregado (N=1.079)**		
Não	29,9	27,3-32,7
Sim	70,1	67,3-72,7
**Comportamentais**		
**Uso de drogas ilícitas (N=1.077)**		
Não	94,1	92,4-95,3
Sim	5,9	4,7-7,5
**Tabagismo (N=1.079)**		
Não	76,2	73,5-78,6
Sim	16,5	14,4-18,8
Ex-fumante	7,3	5,9-9,0
**Consumo de álcool (N=1.086)**		
Não	49,4	46,4-52,3
Sim	50,6	47,7-53,6
**Ciumento (N=1.086)**		
Não	73,3	70,6-75,8
Sim	26,7	24,2-29,4
**Já sentiu medo do parceiro (N=1.086)**		
Não	89,6	87,6-91,3
Sim	10,4	8,7-12,4
**Controlador (N=1.086)**		
Não	76,2	73,6-78,7
Sim	23,8	21,3-26,4
**Recusa a preservativos**		
Não	83,1	80,8-85,3
Sim	16,9	14,7-19,2

*% = Frequência relativa; ^†^IC 95% = Intervalo de confiança de 95%

Pode-se observar na Tabela 2 que houve uma associação entre uma maior frequência de violência psicológica, física e/ou sexual ao longo da vida e as seguintes variáveis: etnia/cor da pele do parceiro e anos de escolaridade do parceiro, renda familiar *per capita*, uso de drogas, tabagismo, parceiros ciumentos, comportamento controlador, sentimento de medo do parceiro e recusa ao uso de preservativos durante a relação sexual (p < 0,05). Além disso, constatou-se que a idade e o consumo de álcool estavam relacionados à violência psicológica e física, e que a variável emprego estava relacionada à violência psicológica.

**Tabela 2:** Prevalência de violência (psicológica, física e/ou sexual) ao longo da vida, de acordo com as características demográficas, socioeconômicas e comportamentais dos parceiros. Vitória, ES, Brasil, 2022

	Violência psicológica		Violência física		Violência sexual	
Características	P* (IC^†^ 95%)	P-valor	P* (IC^†^ 95%)	P-valor	P* (IC^†^ 95%)	P-valor
**Sociodemográficas**						
**Faixa etária (N=1.085)**		<0,001^‡^		0,044^‡^		0,133^‡^
Até 40 anos	52,3 (47,3-57,1)		29,2 (24,9-33,8)		19,6 (16,0-23,8)	
41 anos ou mais	41,2 (37,6-44,9)		23,6 (20,6-26,9)		15,9 (13,3-18,8)	
**Etnia/Cor da pele (N=1.074)**		0,001^§^		<0,001^§^		<0,001^§^
Branco	40,4 (36,2-44,7)		19,2 (16,0-22,9)		11,8 (9,2-14,9)	
Preto	55,1 (48,4-61,7)		33,2 (27,2-39,8)		23,4 (18,2-29,5)	
Pardo	46,6 (41,4-51,8)		30,6 (26,0-35,6)		21,4 (17,4-26,0)	
**Escolaridade (N=1.086)**		0,041^‡^		<0,001^‡^		0,005
Até 8 anos	51,4 (44,8-57,9)		36,4 (30,3-42,9)		23,6 (18,5-29,7)	
9 anos ou mais	43,7 (40,4-47,0)		22,9 (20,2-25,8)		15,6 (13,3-18,2)	
**Renda familiar *per capita* (em tercis) (N=1.067)**		<0,001		<0,001		0,001
1.º tercil - os mais pobres	53,4 (48,3-58,5)		37,5 (32,6-42,6)		23,1 (19,1-27,8)	
2.º tercil	43,2 (38,1-48,4)		21,5 (17,5-26,0)		16,2 (12,7-20,3)	
3.º tercil - os mais ricos	39,1 (34,1-44,4)		18,3 (14,5-22,7)		12,8 (9,6-16,7)	
**Empregado (N=1.079)**		0,006^§^		0,481^§^		0,517^§^
Não	39,0 (33,8-44,4)		24,2 (19,8-29,1)		16,1 (12,5-20,5)	
Sim	48,0 (44,5-51,6)		26,2 (23,2-29,4)		17,7 (15,2-20,6)	
**Comportamentais**						
**Uso de drogas ilícitas (N=1.077)**		<0,001^‡^		<0,001^‡^		0,002^‡^
Não	43,6 (40,6-46,7)		24,1 (21,5-26,8)		16,3 (14,1-18,7)	
Sim	70,3 (58,1-80,2)		46,9 (35,1-59,1)		31,3 (21,1-43,6)	
**Tabagismo (N=1.079)**		<0,001^§^		0,016^§^		0,004^§^
Não	41,2 (37,9-44,6)		23,4 (20,6-26,4)		15,2 (12,9-17,8)	
Sim	60,7 (53,3-67,6)		32,6 (26,1-39,8)		24,2 (18,4-31,0)	
Ex-fumante	51,9 (41,0-62,7)		31,7 (22,4-42,7)		24,1 (15,9-34,7)	
**Consumo de álcool (N=1.086)**		<0,001^‡^		0,005^‡^		0,098^§^
Não	38,6 (34,6-42,8)		21,8 (18,5-25,5)		15,3 (12,5-18,6)	
Sim	51,6 (47,5-55,8)		29,3 (25,6-33,2)		19,1 (16,0-22,6)	
**Ciumento (N=1086)**		<0,001^‡^		<0,001^‡^		<0,001^‡^
Não	40,3 (37,0-43,8)		21,6 (18,9-24,6)		14,2 (11,9-16,8)	
Sim	58,6 (52,9-64,2)		36,6 (31,2-42,3)		25,5 (20,8-30,9)	
**Já sentiu medo do parceiro (N=1.086)**		<0,001^‡^		<0,001^‡^		<0,001^‡^
Não	41,5 (38,5-44,7)		20,9 (18,4-23,5)		14,2 (12,1-16,5)	
Sim	77,0 (68,3-83,8)		66,4 (57,2-74,4)		43,4 (34,5-52,6)	
**Controlador (N=1.086)**		<0,001^‡^		<0,001^‡^		<0,001^‡^
Não	38,7 (35,4-42,0)		21,0 (18,4-23,9)		13,7 (11,5-16,2)	
Sim	66,3 (60,3-71,8)		40,3 (34,5-46,4)		28,7 (23,5-34,5)	
**Recusa a preservativos**		<0,001^‡^		<0,001^‡^		<0,001^‡^
Não	41,6 (38,5-44,9)		23,0 (20,4-25,9)		13,5 (11,4-15,9)	
Sim	62,8 (55,6-69,5)		38,3 (31,5-45,5)		35,5 (28,9-42,7)	

*P = Prevalência; ^†^IC 95% = Intervalo de confiança de 95%; ^‡^Teste exato de Fisher; ^§^Teste do qui-quadrado

Após os ajustes, a Tabela 3 mostrou que as mulheres cujos parceiros eram mais velhos (com 41 anos ou mais) apresentaram uma prevalência 19% menor de violência psicológica. Além disso, esse tipo de abuso foi mais frequente entre mulheres com parceiros de etnia/cor de pele preta (RP: 1,24; IC 95%: 1,06-1,44) e que não tinham emprego (RP: 1,25; IC 95%: 1,08-1,45). Parceiros que fumavam (RP: 1,17; IC 95%: 1,01-1,36), ex-fumantes (RP: 1,30; IC 95%: 1,04-1,61) e aqueles que consumiam álcool (RP: 1,19; IC 95%: 1,04-1,36) representavam as características que apresentavam maior prevalência de violência psicológica quando comparados ao grupo de referência. Mulheres que sentiram medo de seus parceiros (RP: 1,42; IC 95%: 1,24-1,64), com parceiros controladores (RP: 1,48; IC 95%: 1,29-1,70) e com parceiros que se recusavam a usar preservativos durante a relação sexual (RP: 1,24; IC 95%: 1,08-1,42) estavam entre as que haviam sofrido mais violência psicológica, após o controle de possíveis fatores de confusão (p < 0,05).

**Tabela 3:** Análise bruta e ajustada da prevalência da violência psicológica ao longo da vida, de acordo com as características do parceiro. Vitória, ES, Brasil, 2022

Características	Análise bruta		Análise ajustada	
	RP* (IC^†^95%)	P-valor	RP* (IC^†^95%)	P-valor
**Faixa etária**		<0,001		0,002
Até 40 anos	Ref.^‡^		Ref.^‡^	
41 anos ou mais	0,79 (0,69-0,89)		0,81 (0,71-0,93)	
**Etnia/Cor da pele**		0,001		0,029
Branco	Ref.^‡^		Ref.^‡^	
Preto	1,37 (1,16-1,60)		1,24 (1,06-1,44)	
Pardo	1,15 (0,99-1,34)		1,09 (0,94-1,26)	
**Escolaridade**		0,033		0,697
Até 8 anos	1,18 (1,01-1,37)		0,97 (0,82-1,14)	
9 anos ou mais	Ref.^‡^		Ref.^‡^	
**Renda familiar *per capita* (em tercis)**		<0,001		0,232
1.º tercil - os mais pobres	1,37 (1,16-1,61)		1,15 (0,97-1,36)	
2.º tercil	1,10 (0,92-1,32)		1,04 (0,88-1,23)	
3.º tercil - os mais ricos	Ref.^‡^		Ref.^‡^	
**Empregado**		0,009		0,004
Não	0,81 (0,70-0,95)		1,25 (1,08-1,45)	
Sim	Ref.^‡^		Ref.^‡^	
**Uso de drogas ilícitas**		<0,001		0,854
Não	Ref.^‡^		Ref.^‡^	
Sim	1,61 (1,35-1,92)		1,02 (0,82-1,27)	
**Tabagismo**		<0,001		0,016
Não	Ref.^‡^		Ref.^‡^	
Sim	1,47 (1,27-1,70)		1,17 (1,01-1,36)	
Ex-fumante	1,26 (1,00-1,58)		1,30 (1,04-1,61)	
**Consumo de álcool**		<0,001		0,012
Não	Ref.^‡^		Ref.^‡^	
Sim	1,34 (1,17-1,53)		1,19 (1,04-1,36)	
**Ciumento**		<0,001		0,565
Não	Ref.^‡^		Ref.^‡^	
Sim	1,45 (1,28-1,65)		1,04 (0,90-1,22)	
**Já sentiu medo do parceiro**		<0,001		<0,001
Não	Ref.^‡^		Ref.^‡^	
Sim	1,85 (1,64-2,10)		1,42 (1,24-1,64)	
**Controlador**		<0,001		<0,001
Não	Ref.^‡^		Ref.^‡^	
Sim	1,71 (1,52-1,94)		1,48 (1,29-1,70)	
**Recusa a preservativos**		<0,001		0,002
Não	Ref.^‡^		Ref.^‡^	
Sim	1,51 (1,32-1,72)		1,24 (1,08-1,42)	

*RP = Razão de prevalência; ^†^IC 95% = Intervalo de confiança de 95%; ^‡^Ref. = Grupo de referência

Na Tabela 4, os resultados da análise ajustada mostram que as mulheres com parceiros mais velhos (com 41 anos ou mais) apresentaram uma prevalência de violência física ao longo da vida 19% menor quando comparadas às mulheres com parceiros mais jovens (p < 0,05). Mulheres com parceiros pretos sofreram 33% mais violência física, e a prevalência dessa condição foi 73% maior entre aquelas com parceiros mais pobres, ou seja, aqueles pertencentes ao primeiro tercil da renda familiar per capita. Verificou-se que mulheres com parceiros que consumiam álcool apresentaram uma prevalência de sofrer violência física ao longo da vida 1,27 vez maior do que mulheres com parceiros que não consumiam álcool. Vale ressaltar que as participantes que relataram sentir medo de seus parceiros apresentaram maior prevalência de violência física quando comparadas àquelas que não sentiam medo, sendo 2,52 vezes maior. A prevalência de violência física ao longo da vida foi 30% maior entre as mulheres com parceiro controlador do que entre aquelas sem parceiro controlador (p < 0,05).

**Tabela 4:** Análise bruta e ajustada da prevalência de violência física ao longo da vida, de acordo com as características do parceiro. Vitória, ES, Brasil, 2022

	Análise bruta		Análise ajustada	
Características	RP* (IC^†^ 95%)	P-valor	RP* (IC^†^ 95%)	P-valor
**Faixa etária**		0,040		0,037
Até 40 anos	Ref.^‡^		Ref.^‡^	
41 anos ou mais	0,81 (0,66-0,99)		0,81 (0,67-0,99)	
**Etnia/Cor da pele**		<0,001		0,085
Branco	Ref.^‡^		Ref.^‡^	
Preto	1,73 (1,33-2,24)		1,33 (1,02-1,74)	
Pardo	1,59 (1,25-2,02)		1,25 (0,98-1,59)	
**Escolaridade**		<0,001		0,590
Até 8 anos	1,59 (1,28-1,97)		1,06 (0,85-1,33)	
9 anos ou mais	Ref.^‡^		Ref.^‡^	
**Renda familiar *per capita* (em tercis)**		<0,001		<0,001
1.º tercil - os mais pobres	2,05 (1,58-2,66)		1,73 (1,34-2,24)	
2.º tercil	1,17 (0,87-1,58)		1,12 (0,84-1,50)	
3.º tercil - os mais ricos	Ref.^‡^		Ref.^‡^	
**Empregado**		0,484		
Não	0,92 (0,73-1,16)		-	-
Sim	^‡^Ref.		-	-
**Uso de drogas ilícitas**		<0,001		0,323
Não	Ref.^‡^		Ref.^‡^	
Sim	1,95 (1,47-2,58)		1,18 (0,85-1,63)	
**Tabagismo**		0,013		0,072
Não	Ref.^‡^		Ref.^‡^	
Sim	1,40 (1,09-1,78)		0,94 (0,74-1,21)	
Ex-fumante	1,35 (0,96-1,92)		1,43 (1,03-2,00)	
**Consumo de álcool**		0,005		0,017
Não	Ref.^‡^		Ref.^‡^	
Sim	1,34 (1,09-1,65)		1,27 (1,04-1,54)	
**Ciumento**		<0,001		0,620
Não	Ref.^‡^		Ref.^‡^	
Sim	1,69 (1,38-2,07)		1,06 (0,84-1,33)	
**Já sentiu medo do parceiro**		<0,001		<0,001
Não	Ref.^‡^		Ref.^‡^	
Sim	3,18 (2,66-3,81)		2,52 (2,02-3,13)	
**Controlador**		<0,001		0,019
Não	Ref.^‡^		Ref.^‡^	
Sim	1,92 (1,57-2,34)		1,30 (1,04-1,62)	
**Recusa a preservativos**		<0,001		0,079
Não	Ref.^‡^		Ref.^‡^	
Sim	1,66 (1,33-2,07)		1,22 (0,98-1,52)	

*RP = Razão de prevalência; ^†^IC 95% = Intervalo de confiança de 95%; ^‡^Ref. = Grupo de referência

A Tabela 5 mostra que as mulheres com parceiros pretos e pardos apresentaram uma prevalência de violência sexual ao longo da vida 75% e 69% maior, respectivamente, quando comparadas às mulheres com parceiros brancos. Além disso, as participantes que relataram sentimentos de medo em relação aos seus parceiros (RP: 1,97; IC 95%: 1,48-2,63) e que os consideravam controladores (RP: 1,43; IC 95%: 1,09-1,86) também apresentaram maior prevalência desse tipo de abuso. A recusa em usar preservativo foi associada a 2,18 vezes mais ocorrências de violência sexual cometida por um parceiro íntimo (p < 0,05).

**Tabela 5:** Análise bruta e ajustada da prevalência de violência sexual ao longo da vida, de acordo com as características do parceiro. Vitória, ES, Brasil, 2022

	Análise bruta		Análise ajustada	
Características	RP* (IC^†^ 95%)	P-valor	RP* (IC^†^ 95%)	P-valor
**Faixa etária**		0,116		0,190
Até 40 anos	Ref.^‡^		Ref.^‡^	
41 anos ou mais	0,81 (0,62-1,05)		0,84 (0,64-1,09)	
**Etnia/Cor da pele**		<0,001		0,001
Branco	Ref.^‡^		Ref.^‡^	
Preto	1,99 (1,41-2,79)		1,75 (1,26-2,43)	
Pardo	1,82 (1,33-2,49)		1,69 (1,25-2,29)	
**Escolaridade**		0,004		0,750
Até 8 anos	1,52 (1,14-2,01)		1,05 (0,77-1,43)	
9 anos ou mais	Ref.^‡^		Ref.^‡^	
**Renda familiar *per capita* (em tercis)**		0,001		0,555
1.º tercil - os mais pobres	1,81 (1,30-2,53)		1,20 (0,85-1,72)	
2.º tercil	1,27 (0,88-1,82)		1,08 (0,75-1,54)	
3.º tercil - os mais ricos	Ref.^‡^		Ref.^‡^	
**Empregado**		0,519		
Não	0,91 (0,68-1,22)		-	-
Sim	Ref.^‡^		-	-
**Uso de drogas ilícitas**		0,001		0,421
Não	Ref.^‡^		Ref.^‡^	
Sim	1,92 (1,30-2,83)		1,20 (0,77-1,86)	
**Tabagismo**		0,004		0,171
Não	Ref.^‡^		Ref.^‡^	
Sim	1,59 (1,17-2,16)		1,18 (0,87-1,59)	
Ex-fumante	1,58 (1,04-2,42)		1,47 (0,95-2,25)	
**Consumo de álcool**		0,099		0,493
Não	Ref.^‡^		Ref.^‡^	
Sim	1,25 (0,96-1,62)		1,10 (0,84-1,44)	
**Ciumento**		<0,001		0,804
Não	Ref.^‡^		Ref.^‡^	
Sim	1,80 (1,39-2,33)		1,04 (0,78-1,38)	
**Já sentiu medo do parceiro**		<0,001		<0,001
Não	Ref.^‡^		Ref.^‡^	
Sim	3,06 (2,35-3,97)		1,97 (1,48-2,63)	
**Controlador**		<0,001		0,009
Não	Ref.^‡^		Ref.^‡^	
Sim	2,10 (1,62-2,72)		1,43 (1,09-1,86)	
**Recusa a preservativos**		<0,001		<0,001
Não	Ref.^‡^		Ref.^‡^	
Sim	2,63 (2,04-3,40)		2,18 (1,68-2,83)	

*RP = Razão de prevalência; ^†^IC 95% = Intervalo de confiança de 95%; ^‡^Ref. = Grupo de referência

## Discussão

O estudo revelou uma associação entre as características sociodemográficas e comportamentais dos parceiros íntimos e a ocorrência de violência contra as mulheres ao longo da vida. Verificou-se que mulheres com parceiros que apresentavam comportamento controlador, comportamento ciumento, recusavam-se a usar preservativos durante a relação sexual, eram fumantes ou ex-fumantes, consumiam álcool, não tinham emprego, pertenciam à etnia/cor de pele preta e ao primeiro tercil da renda familiar *per capita* (os mais pobres) apresentavam maior prevalência de violência (psicológica, física e/ou sexual) ao longo da vida. Os resultados estão em consonância com estudos nacionais realizados entre mulheres usuárias de atenção primária^3^ e com puérperas em uma maternidade de risco habitual^16^.

Mulheres com parceiros mais jovens, com idade até 40 anos, apresentaram maior prevalência de violência psicológica e física, após ajustes. Os resultados deste estudo corroboram um estudo nacional realizado em 2003 com 251 mulheres usuárias de serviços de saúde em Porto Alegre, Rio Grande do Sul, que demonstrou uma associação entre a ocorrência de violência psicológica e física e a idade do parceiro inferior a 30 anos^17^. Um estudo internacional mostrou que mulheres com parceiros mais velhos (com 60 anos ou mais) tinham 43% menos chances de sofrer violência por parceiro íntimo do que aquelas com parceiros mais jovens (18-29 anos)^4^.

A maior prevalência de violência física foi observada entre mulheres cujos parceiros pertenciam ao 1º tercil de renda (os mais pobres), em comparação com aquelas cujos parceiros pertenciam ao tercil mais rico. É fundamental contextualizar que a associação entre maior pobreza e maior reconhecimento da violência contra as mulheres não deve ser interpretada como uma relação causal direta, mas como um reflexo de condições estruturais. Contextos de pobreza frequentemente envolvem maior estresse financeiro, moradia precária, menor acesso a redes de apoio e serviços de proteção, fatores que podem intensificar conflitos e reduzir alternativas para resolução não violenta. Além disso, a violência perpetrada por homens pobres tende a ser mais visível e menos tolerada socialmente, enquanto comportamentos semelhantes em grupos socioeconômicos mais elevados podem ser normalizados ou ocultados, afetando o reconhecimento e a denúncia da violência^18^.

Outras características do agressor associadas a maiores probabilidades de cometer violência psicológica, física e sexual foram a etnia/cor de pele preta. A realidade socioeconômica e racial do Brasil mostra que os homens pretos brasileiros apresentam um nível de escolaridade mais baixo e ocupam cargos menos prestigiados quando comparados aos homens brancos^19^. Homens e mulheres com menor escolaridade estão mais expostos a sofrer violência, sendo que 91,8% das vítimas de homicídio são homens e 75,7% são homens pretos^20^.

É importante considerar que esse resultado pode refletir um preconceito racial na percepção de comportamentos violentos, em que estereótipos associam homens pretos a uma maior agressividade, levando a um reconhecimento mais rápido ou mais intenso da violência quando o agressor é preto do que quando ele é branco, mesmo em situações semelhantes - um fenômeno que já foi observado em estudos sobre como a raça influencia julgamentos de violência e culpabilidade em casos de violência por parceiros íntimos, por exemplo, pesquisas sobre percepções de violência por parceiros íntimos e decisões de júris em cenários racialmente diferenciados indicam que a raça do agressor altera a forma como a violência é avaliada^21^.

Além disso, as mulheres cujos parceiros não trabalhavam apresentaram uma prevalência de violência psicológica ao longo da vida mais elevada. Os homens sem emprego estável tendem a exercer um maior controle coercitivo sobre suas parceiras, tendo em vista que o estresse econômico pode ser um importante fator preditivo de violência^5^.

Ficou evidenciado que as mulheres que relataram sentir medo de seus parceiros apresentaram maior prevalência de violência (psicológica, física e sexual) ao longo de suas vidas. Relacionamentos interpessoais violentos são frequentemente marcados pelo medo e por comportamentos controladores entre os parceiros^1^, portanto, o comportamento violento masculino capaz de justificar o medo sofrido por essas mulheres é um fator relevante para as consequências físicas e psicológicas antes e depois da ocorrência da violência. Tal comportamento masculino opressivo está relacionado à cultura patriarcal e à sua estrutura, na qual homens e mulheres não podem ser separados, reproduzindo assim o papel de subordinação das mulheres aos homens^22^.

É preciso levar em conta as diferentes esferas que podem desencadear o medo nas mulheres em situações de violência doméstica, e o medo surge como um fator paralisante, incapacitando a reação e a quebra do ciclo^8^. Como já discutido, o medo também pode ser resultado de uma longa série de ameaças por parte do agressor, além de ser potencialmente prejudicial a curto e longo prazo, intensificando-se com a sensação de impunidade com que o Estado responde à violência^8^. Ele também pode basear-se em ameaças à segurança da própria vida das mulheres e à vida de pessoas próximas a elas, como filhos e familiares^22^.

No que diz respeito ao uso de preservativos, o estudo constatou que as mulheres cujos parceiros se recusavam a usar preservativos apresentavam maior prevalência de violência psicológica e sexual. Em consonância com os resultados deste estudo, a literatura reafirma as evidências apontadas^3^ ^,^ ^16^ ^-^ ^17^. O resultado, após ajustes, encontrado neste estudo para a violência sexual foi o mesmo observado em um estudo com mulheres usuárias de serviços de atenção primária à saúde na mesma cidade^3^. O estudo mostrou que mulheres com parceiros que se recusavam a usar preservativos tinham nove vezes mais chances de sofrer violência sexual, mesmo durante a gravidez^16^. A dificuldade de negociar tanto o uso de preservativos quanto de outros métodos contraceptivos aponta para a fragilidade do poder de decisão das mulheres sobre sua própria saúde sexual e reprodutiva, evidenciando um contexto em que esse poder pertence ao parceiro^3^ ^,^ ^16^ ^,^ ^23^.

Além do papel da dominação masculina na decisão de usar preservativos, há também a questão da dominação sobre a saúde sexual das mulheres. Sabe-se que os homens que compreendem que o uso do preservativo previne doenças como o HIV também exercem uma influência mais positiva nas suas relações sexuais com as mulheres, em comparação com os homens que não compreendem os métodos de proteção sexual^24^. Além disso, um estudo realizado no Haiti mostrou que a recusa das mulheres em ter relações sexuais estava associada à violência física e a taxas concomitantes mais baixas de uso de preservativos^24^.

No que diz respeito ao comportamento controlador masculino, as mulheres que vivem em relacionamentos violentos podem sofrer afastamento social da família e dos amigos, uma vez que vivem sob vigilância constante e imposição de regras que violam os direitos humanos e prejudicam sua qualidade de vida. Além do controle social, as mulheres têm poder limitado em situações de relacionamento sexual, o que dificulta a negociação do uso de preservativos e as coloca em risco de sofrer violência^1^ ^,^ ^23^.

Além da desigualdade de gênero e da desigualdade nas relações de poder no âmbito da estrutura social e das relações interpessoais, a desigualdade social também é um fator importante para que o ciclo de violência não seja rompido. Fatores como a dependência financeira^25^, o baixo nível de escolaridade^23^, as preocupações com o futuro dos filhos e a dependência emocional são determinantes para que as mulheres permaneçam no ciclo de violência^8^ ^,^ ^26^. Outro fator relevante para a vulnerabilidade de permanecer no contexto de violência está relacionado à experiência de outros tipos de violência doméstica ao longo da vida, bem como a um contexto familiar violento que pode influenciar a naturalização da violência^27^.

Este estudo constatou uma maior prevalência de violência psicológica contra mulheres cometida por homens fumantes ou ex-fumantes, resultado também observado em um estudo realizado na cidade de Shenzhen, na China^28^. Isso provavelmente se deve a fatores associados ao tabagismo, como estresse, impulsividade e uso concomitante de álcool e outras substâncias, que já são conhecidos como fatores de risco para a violência^28^ ^-^ ^29^.

Neste estudo, o consumo de álcool pelo parceiro íntimo foi associado a uma maior prevalência de violência psicológica e física. O álcool é uma substância psicoativa, e seu consumo afeta as conexões e vias neurais. Além disso, dependendo da quantidade consumida, os efeitos podem ser intensificados, com possíveis consequências que levam ao comprometimento da atenção, da cognição e da destreza; ao aumento da impulsividade; a um perfil agressivo; à perda de controle comportamental e à exacerbação das reações^30^. Portanto, o consumo de álcool pode aumentar o risco de violência devido à possibilidade de conflito e à externalização de comportamentos violentos, expressando um lado impulsivo e agressivo dos parceiros que possuem uma construção social com o machismo e o patriarcado arraigados^3^ ^,^ ^5^ ^,^ ^16^.

No Brasil, os homens são os principais consumidores de álcool em excesso quando comparados às mulheres^30^. O risco atribuível e os custos hospitalares relacionados à violência são elevados, e o custo médio total da violência interpessoal entre 2010 e 2018 atribuível ao álcool pode ser estimado em mais de 17 milhões de dólares^26^. Entende-se que o fenômeno em estudo é multidimensional. No entanto, o Brasil é um dos países que tem apresentado lento progresso na implementação de políticas públicas para combater o uso nocivo do álcool, sem articulação intersetorial voltada para a redução do risco de violência^31^.

A ocorrência de violência ao longo da vida pode causar diversas consequências físicas, psicológicas e sociais, a curto e longo prazo, para as mulheres com histórico de violência; além disso, o medo, aliado à dependência financeira e emocional, pode dificultar a quebra do ciclo de violência^1^. Os profissionais de saúde são, portanto, um ponto crítico de comunicação para essas pessoas^32^. No entanto, para prestar atendimento eficaz e eficiente às mulheres em situações de violência, é essencial que os profissionais de saúde desenvolvam sua perspectiva sobre esse grave problema de saúde pública, que abrange diferentes dimensões da vida humana (social, espiritual, física, mental e biológica). É também necessário que essa perspectiva profissional tenha um caráter interprofissional e intersetorial, para que os principais encaminhamentos sejam feitos de acordo com as reais necessidades das mulheres atendidas^33^. Além disso, é importante destacar a notificação obrigatória que deve ser realizada por esses profissionais em casos ou suspeitas de violência. Isso se tornou uma ferramenta fundamental para a vigilância epidemiológica e a formulação de políticas públicas de prevenção e intervenção^34^.

Os resultados deste estudo são de grande relevância para a enfermagem clínica e outros profissionais de saúde, especialmente no atendimento a mulheres em consultas ginecológicas e obstétricas, pois reforçam a importância de conhecer o perfil de seus parceiros, considerando que certas características podem levar a um maior risco de violência. Os resultados, portanto, reforçam a necessidade de uma coleta de dados mais completa e holística, bem como a necessidade de denunciar esse problema e incluir as mulheres na rede de atendimento e proteção.

Este é o primeiro estudo de base populacional a descrever o perfil do parceiro atual entre mulheres que vivem em uma capital da região sudeste do país. A violência foi medida por meio de um instrumento validado e foi garantida às mulheres a privacidade para responder a todo o questionário. O estudo contribui para a literatura nacional e internacional sobre o perfil dos parceiros de mulheres com maior prevalência de violência ao longo da vida, que apresenta várias lacunas; assim, as informações apresentadas podem apoiar o planejamento e a implementação de políticas públicas intersetoriais para combater a violência contra as mulheres.

É preciso mencionar algumas limitações, como a causalidade reversa, uma vez que se trata de um estudo transversal e a temporalidade da relação fica comprometida, tornando muito importante a realização de estudos longitudinais. O viés de desejabilidade social pode levar as mulheres vítimas de violência doméstica a ajustar seus relatos para se adequarem às normas sociais ou às expectativas do pesquisador. Isso pode resultar na minimização da gravidade da violência, por meio da omissão de episódios ou da descrição do agressor sob uma luz menos negativa, especialmente quando há medo de julgamento ou estigmatização. Além disso, o sentimento de culpa, a dependência emocional ou econômica podem influenciar respostas mais defensivas em relação ao agressor. Assim, esse viés pode comprometer a precisão e a integridade dos dados sobre a violência sofrida. Pelas razões já mencionadas, também pode haver confusão residual.

## Conclusão

Os resultados mostraram uma associação entre a violência contra as mulheres e as características socioeconômicas e comportamentais de seus parceiros íntimos. Sabe-se que o fenômeno da violência é complexo e tem múltiplas causas, mas este estudo demonstra que essas características, especialmente as comportamentais dos parceiros íntimos, apresentam uma associação mais forte com a ocorrência de violência. Esses dados apontam um caminho para medidas de intervenção baseadas em evidências, uma vez que o comportamento pode ser modificado desde que sejam aplicadas intervenções eficientes. Os resultados destacam a importância da ação intersetorial para promover uma cultura de paz, combater a violência e a desigualdade social e de gênero, bem como a necessidade de reduzir o consumo de álcool, tabaco e outras drogas.

## Data Availability

Os conjuntos de dados relacionados a este artigo estarão disponíveis mediante solicitação ao autor correspondente.

# Translation: Asociación entre violencia contra las mujeres y características de sus parejas sexuales: estudio poblacional realizado en Vitória, Espírito Santo^*^

**DOI:** 10.1590/1518-8345.7809.4966

**Authors:** Franciéle Marabotti Costa Leite, Julya de Almeida Polverine, Dherik Fraga Santos, Bruna Venturin

**Affiliations:** 1 Universidade Federal do Espírito Santo, Vitória, ES, Brasil.; 2 Scholarship holder at the Conselho Nacional de Desenvolvimento Científico e Tecnológico (CNPq), Brasil.; 3 Universidade Federal do Espírito Santo, Departamento de Enfermagem, Vitória, ES, Brasil.; 4 Universidade Federal de Catalão, Departamento de Medicina, Catalão, GO, Brasil.; 5 Universidade Federal de Pelotas, Pelotas, RS, Brasil.

**Keywords:** Violencia, Violencia de Pareja, Violencia Doméstica, Violencia contra la Mujer, Factores de Riesgo, Estudios Transversales.

## Abstract

**Objetivo:**

analizar la asociación entre la violencia contra las mujeres y las características sociodemográficas y conductuales de sus parejas sexuales.

**Método:**

estudio transversal realizado con 1086 mujeres de al menos 18 años de edad que hubieren tenido pareja sexual en los últimos 24 meses. Se utilizó el instrumento sobre violencia contra las mujeres de la Organización Mundial de la Salud para clasificar casos de violencia psicológica, física y sexual. Se recolectaron características sociodemográficas y conductuales de las parejas sexuales. Los resultados se presentaron como razones de prevalencia, con sus respectivos intervalos de confianza del 95%.

**Resultados:**

las mujeres cuyas parejas tenían al menos 41 años de edad presentaron una prevalencia 19% más baja de violencia psicológica y física. Se detectó una prevalencia más alta de dicho abuso entre mujeres con pareja que eran de raza negra y consumían alcohol. La violencia psicológica fue un 25%, 17% y 30% más elevada, respectivamente, entre las mujeres cuyas parejas no tenían empleo pago, fumaban o eran ex-fumadores. La violencia física fue un 73% más frecuente entre las participantes cuyas parejas pertenecían al primer tercil en materia de ingresos familiares per cápita. La victimización psicológica, física o sexual fue más alta entre las mujeres que tenían miedo de sus parejas (RP: 1,42; IC95%: 1,24-1,64; RP: 2,52; IC95%: 2,02-3,13; RP: 1,97; IC95%: 1,48-2,63) y cuyas parejas eran controladoras (RP: 1,48; IC95%: 1,29-1,70; RP: 1,30; IC95%: 1,04-1,62; RP: 1,43; IC 95%: 1,09-1,86).

**Conclusión:**

las características sociodemográficas y conductuales de las parejas intensifican la vulnerabilidad de las mujeres ante la violencia.

## Destacados

**(1)**Mayor prevalencia de violencia contra mujeres que tienen parejas controladoras.

**(2)**Las mujeres que alguna vez sintieron miedo de sus parejas presentaron mayor prevalencia de casos de violencia.

**(3)**El hecho de que las parejas consuman alcohol estuvo asociado a mayor frecuencia de casos de violencia.

**(4)**Rehusarse a utilizar preservativos estuvo asociado a mayor prevalencia de casos de violencia.

**(5)**Se deben fomentar políticas de igualdad de género y abordar las formas de violencia contra las mujeres.

## Introducción

La Organización de las Naciones Unidas define “violencia contra las mujeres” como cualquier acción violenta sobre la base del género que deriva o tiene probabilidades de derivar en sufrimiento físico, sexual o mental en ellas^1^. En consecuencia, la violencia de pareja (VP) se caracteriza como un agente habitual que actúa en casos de violencia y se relaciona con las víctimas, en la figura de pareja sexual. Además, la VP es una de las formas más prevalentes de violencia contra las mujeres e implica acciones como bofetadas, arrojar objetos que pueden lastimar a la otra persona, empujones y patadas^2^. Las estimaciones globales de la Organización Mundial de la Salud (OMS) indican que 1 de cada 3 mujeres sufrió o sufrirá violencia de pareja a lo largo de su vida, lo que representa aproximadamente el 35% de la población femenina^1^. La OMS también señala que el 38% de los femicidios son las parejas sexuales de las mujeres^1^.

En todo el mundo, las mujeres denuncian sufrir violencia perpetrada por sus parejas. De acuerdo con un estudio transversal desarrollado con usuarias de atención primaria en Vitória, ES (Brasil) que tenían pareja sexual, la violencia perpetrada por las parejas sexuales se asoció con desempleo, rehusarse a utilizar preservativos en las relaciones sexuales, conductas controladoras, consumo de alcohol, hábito de fumar y parejas con menos de 8 años de estudio^3^. En Wuhan (China), también se observó que las parejas que fumaban (33%) y consumían alcohol (35%) eran más propensas a ejercer violencia doméstica que aquellas que no fumaban (23,89%) ni bebían (24,21%)^4^. En Europa, la prevalencia en términos de mujeres que han sufrido violencia de pareja en algún momento de sus vidas es del 51,7%; además, el estudio también concuerda con el perfil de parejas sexuales agresivas: personas de menos 40 años de edad y desempleadas o con salarios más bajos que sus parejas^5^.

La violencia contra las mujeres acarrea consecuencias a corto, mediano y largo plazo para la salud física y mental de las víctimas^6^. Con respecto a la salud de las mujeres en situaciones de violencia de pareja física y/o sexual, se registra un alto índice de problemas de salud de acuerdo con la OMS, a saber: las víctimas son dos veces más propensas a sufrir abortos y a padecer depresión, además de tener 1,5 veces más chances de contraer VIH en comparación con aquellas que no sufren casos de violencia^1^. La exposición a la violencia también puede derivar en cefaleas, trastornos sexuales, fobias y temores prolongados^7^ ^-^ ^8^.

De acuerdo con un estudio transversal, las mujeres que sufren VP tienen más probabilidades de retrasarse con sus exámenes citopatológicos periódicos (Papanicolaou), con una demora del 64% en el caso de víctimas de violencia sexual, mientras que la demora se duplica en quienes padecen violencia física^9^. Tal como se ha demostrado, la violencia de pareja continúa amenazando la salud física y mental de las mujeres en todo el mundo. Pese a la mayor cantidad de trabajos de investigación sobre la incidencia de la violencia de pareja y su relación con el perfil demográfico, socioeconómico y sanitario de las mujeres, pocos estudios investigan las características de las parejas sexuales^10^ ^-^ ^11^.

En una revisión sistemática sobre el perfil de las personas que perpetran violencia contra las mujeres ya se ha destacado la importancia de analizar las características de los atacantes, proporcionando información más allá de meros datos sociodemográficos y abordando las conductas y formas de violencia psicológica adoptadas por los agresores^10^ ^-^ ^11^. El vacío es importante; llenarlo puede mejorar la efectividad de las intervenciones en relación con este problema. Hasta el momento no se ha realizado ningún trabajo de investigación sobre este tema en la ciudad donde se desarrolló el estudio; además, se han reportado pocos estudios poblacionales sobre el mismo tópico en otros lugares de Brasil^12^. Esta investigación ayudará a comprender mejor el fenómeno.

Cabe destacar que en este estudio se hipotetiza que las conductas de las parejas (como control y consumo de sustancias) predicen la violencia. En este contexto, el objetivo de este estudio fue analizar la asociación entre la violencia contra las mujeres y las características sociodemográficas y conductuales de sus parejas sexuales.

## Método

### Tipo de estudio

Estudio transversal, analítico y poblacional basado en los lineamientos estipulados en *Strengthening the Reporting of Observational Studies in Epidemiology*(STROBE).

### Contexto

Este estudio se realizó en la ciudad de Vitória, Espírito Santo (Brasil). La ciudad de Vitória (capital del estado de Espírito Santo) tenía una población estimada de 369.534 habitantes en el año 2021, en un territorio de 97.123 km^2^ y con un Índice de Desarrollo Humano Municipal de 0,845. Tiene 79 vecindarios y seis áreas regionales de salud^13^. De acuerdo con datos del Censo 2010, la población femenina (163.853 mujeres) representaba el 49,98% del total de habitantes^14^.

### Participantes

La población del estudio estuvo compuesta por mujeres de al menos 18 años de edad que tenían o habían tenido pareja sexual en los últimos 24 meses antes de la entrevista. En este estudio, “pareja sexual” se define como una pareja o ex-pareja y/o novio actual, con la condición de que mantuvieran relaciones sexuales, independientemente de si se tratara o no de una relación marital formal o no formal.

### Criterios de selección

El criterio de exclusión se definió de la siguiente manera: mujeres imposibilitadas de comprender información o de comunicarse debido a deficiencias intelectuales o sensoriales y que, en consecuencia, no pudieran responder los instrumentos utilizados para recolectar los datos de la investigación.

### Definición de la muestra

Se adoptó un proceso de muestreo multietapas. Las unidades de muestreo primarias fueron los distritos censales de la ciudad de Vitória provistos por el Censo 2010 desarrollado por el Instituto Brasileño de Geografía y Estadística (IBGE).

A fin de calcular el tamaño de la muestra, se consideró una prevalencia estimada de violencia de pareja del 50% para maximizar la muestra, con nivel de confianza del 95% y error aceptable del 5%. Para estudiar las asociaciones entre factores de riesgo y desenlaces, se especificó un nivel de confianza del 95% y un poder estadístico del 80%. También se consideró una relación exposición/no exposición de 1:1. A este valor se le sumó un 10% por pérdidas y un 30% por factores de confusión, llegando así al tamaño de muestra necesario: 1100 mujeres.

### Variables del estudio

Las variables independientes relacionadas con las características sociodemográficas de las parejas de las mujeres fueron las siguientes: grupo etario en años completos, grupo étnico/color de piel, años de estudio, ingresos familiares per cápita en terciles y tener empleo. Las variables conductuales analizadas fueron consumo de drogas ilícitas, consumo de tabaco, consumo de alcohol, sentir miedo de la pareja, parejas celosas, parejas controladoras y rehusarse a utilizar preservativos.

Las variables dependientes de este estudio fueron los tres tipos de violencia contra las mujeres (psicológica, sexual y física) perpetrados por parejas sexuales a lo largo de la vida. Se consideró que cada uno de ellos estaba presente cuando una mujer respondía “Sí” a uno de los ítems correspondientes a cada tipo de violencia (psicológica, física o sexual).

### Instrumentos utilizados para recolectar la información

Para obtener las variables independientes se desarrolló un instrumento que contenía características sociodemográficas y conductuales. La variable “Grupo étnico/Color de piel” fue autoinformada de acuerdo con el IBGE: blanco, negro y mestizo.

Se utilizó la versión corta del cuestionario original de la OMS sobre violencia contra las mujeres para identificar los desenlaces. Este cuestionario está validado en Brasil y su propósito es detectar las diferentes formas de violencia contra las mujeres en sus dominios: Psicológica, Física y Sexual. Este instrumento tiene 13 preguntas y presenta alta consistencia interna, tal como lo indicado por los coeficientes de Cronbach (promedio de 0,88)^15^.

### Recolección de datos

Se realizó un estudio piloto en diciembre de 2021. Los datos fueron recopilados de enero a mayo de 2022 por un equipo de entrevistadoras debidamente capacitadas. El equipo de investigación también incluía supervisores de campo responsables de monitorear a los investigadores de campo en relación con su adhesión al cronograma de recopilación de datos, identificando y corrigiendo fallas en dicho proceso y/o inconsistencias en los datos. La información recolectada en el estudio piloto no formó parte de la muestra final de la investigación. El trabajo de campo comenzó una vez analizados los datos obtenidos en el estudio piloto. Las entrevistas se realizaron en las casas de las participantes, en un lugar reservado y respetando su privacidad y confidencialidad. Todas estas medidas se tomaron para minimizar posibles sesgos.

### Procesamiento y análisis de los datos

El análisis descriptivo se realizó presentando frecuencias relativas. Se utilizó una prueba de heterogeneidad y la prueba exacta de Fisher en el análisis bivariado, de acuerdo con las presunciones. Se evaluó la multicolinealidad entre las variables explicativas utilizando el factor de inflación de la varianza, particularmente debido a la potencial superposición entre las características socioeconómicas, sin encontrar evidencia de ella, tal como se observó con el Factor de Inflación de la Varianza (FIV) (considerado multicolineal cuando es mayor que 5). Se empleó regresión Poisson con varianza robusta para estimar las razones de prevalencia (RP) ajustadas y no ajustadas y sus respectivos intervalos de confianza del 95% (IC95%). Inicialmente, las variables asociadas a los desenlaces en estudio (p<0,20) fueron incluidas en el modelo para considerar posibles factores de confusión. Se utilizó el método *backward selection* para excluir variables. Se adoptó un nivel de significancia del 5% (p<0,05) en el modelo final. Los análisis estadísticos se realizaron en el programa estadístico Stata^®^, versión 15.1.

### Aspectos éticos

El estudio fue aprobado por el Comité de Ética en Investigación de la Universidad Federal de Espírito Santo, conforme al informe número 2.819.597. Todas las entrevistadas firmaron un Formulario de Consentimiento Informado; también se les informaron los objetivos del estudio y sus posibles riesgos y beneficios.

## Resultados

Con respecto a la Tabla 1, cabe destacar que la mayoría de las parejas tenía al menos 41 años de edad (63,3%), que aproximadamente el 48% era de raza blanca, que casi el 80% tenía al menos 9 años de estudio, que el 34,0% se encontraba en el primer tercil en cuanto a ingresos familiares y que el 70,1% tenía empleo. En cuanto a las características conductuales, aproximadamente el 6% consumía drogas ilícitas, el 16,5% fumaba y casi el 51% consumía alcohol. Según las mujeres, aproximadamente el 27% de sus parejas eran celosas y el 24%, controladoras; además, el 10,4% declaró haber sentido miedo de sus parejas masculinas. Con respecto al uso de preservativos, el 16,9% de las parejas sexuales ya se había rehusado a utilizarlos durante las relaciones sexuales.

**Tabla 1:** Características sociodemográficas y conductuales correspondientes a las parejas de las mujeres que viven en la ciudad de Vitória, ES, Brasil, 2022 (N=1086)

Características	%*	IC95%^†^
Sociodemográficas		
Grupo etario (N=1085)		
Hasta 40 años	36,7	33,9-39,6
Al menos 41 años	63,3	60,4-66,1
**Grupo étnico/Color de piel (N=1074)**		
Blanco	47,5	44,5-50,5
Negro	19,9	17,6-22,4
Mestizo	32,6	29,8-35,5
**Años de estudio (N=1086)**		
Hasta 8	20,3	18,0-22,8
Al menos 9	79,7	77,2-82,0
**Ingresos familiares per cápita (terciles) (N=1067)**		
1^er^ tercil - El nivel más pobre	34,0	31,2-36,9
2^o^ tercil	33,7	30,9-36,5
3^er^ tercil - El nivel más rico	32,3	29,6-35,2
**Tienen empleo (N=1079)**		
No	29,9	27,3-32,7
Sí	70,1	67,3-72,7
**Conductuales**		
**Consumen drogas ilícitas (N=1077)**		
No	94,1	92,4-95,3
Sí	5,9	4,7-7,5
**Consumen tabaco (N=1079)**		
No	76,2	73,5-78,6
Sí	16,5	14,4-18,8
Ex-fumadores	7,3	5,9-9,0
**Consumen alcohol (N=1086)**		
No	49,4	46,4-52,3
Sí	50,6	47,7-53,6
**Parejas celosas (N=1086)**		
No	73,3	70,6-75,8
Sí	26,7	24,2-29,4
**Ya sintieron miedo de sus parejas (N=1086)**		
No	89,6	87,6-91,3
Sí	10,4	8,7-12,4
**Parejas controladoras (N=1086)**		
No	76,2	73,6-78,7
Sí	23,8	21,3-26,4
**Se rehúsan a usar preservativos**		
No	83,1	80,8-85,3
Sí	16,9	14,7-19,2

*% = Frecuencia relativa; ^†^IC95% = Intervalo de confianza del 95%

En la Tabla 2 puede verse que hubo una asociación entre mayor frecuencia de violencia psicológica, física y/o sexual a lo largo de la vida de las mujeres y las siguientes variables: grupo étnico/color de piel y años de estudio de la pareja; ingresos familiares per cápita, consumo de drogas, fumar, parejas celosas, parejas controladoras; sentir miedo de la pareja; y rehusarse a utilizar preservativos durante las relaciones sexuales (p<0,05). También se descubrió que la edad y el consumo de alcohol estuvieron relacionados con violencia psicológica y física y que la variable “Tienen empleo” se relacionó con la violencia psicológica.

**Tabla 2:** Prevalencia de casos de violencia (psicológica, física y/o sexual) a lo largo de la vida de acuerdo con las características demográficas, socioeconómicas y conductuales de las parejas. Vitória, ES, Brasil, 2022

Características	Violencia psicológica			Violencia física		Violencia sexual	
	P* (IC95%^†^)	Valor p	P* (IC95%^†^)	Valor p	P* (IC95%^†^)	Valor p
**Sociodemográficas**						
**Grupo etario (N=1085)**		<0,001^‡^		0,044^‡^		0,133^‡^
Hasta 40 años	52,3 (47,3-57,1)		29,2 (24,9-33,8)		19,6 (16,0-23,8)	
Al menos 41 años	41,2 (37,6-44,9)		23,6 (20,6-26,9)		15,9 (13,3-18,8)	
**Grupo étnico/ Color de piel (N=1074)**		0,001^§^		<0,001^§^		<0,001^§^
Blanco	40,4 (36,2-44,7)		19,2 (16,0-22,9)		11,8 (9,2-14,9)	
Negro	55,1 (48,4-61,7)		33,2 (27,2-39,8)		23,4 (18,2-29,5)	
Mestizo	46,6 (41,4-51,8)		30,6 (26,0-35,6)		21,4 (17,4-26,0)	
**Años de estudio (N=1086)**		0,041^‡^		<0,001^‡^		0,005
Hasta 8	51,4 (44,8-57,9)		36,4 (30,3-42,9)		23,6 (18,5-29,7)	
Al menos 9	43,7 (40,4-47,0)		22,9 (20,2-25,8)		15,6 (13,3-18,2)	
**Ingresos familiares per cápita (en terciles) (N=1067)**		<0,001		<0,001		0,001
1^er^ tercil - El nivel más pobre	53,4 (48,3-58,5)		37,5 (32,6-42,6)		23,1 (19,1-27,8)	
2^o^ tercil	43,2 (38,1-48,4)		21,5 (17,5-26,0)		16,2 (12,7-20,3)	
3^er^ tercil - El nivel más rico	39,1 (34,1-44,4)		18,3 (14,5-22,7)		12,8 (9,6-16,7)	
**Tienen empleo (N=1079)**		0,006^§^		0,481^§^		0,517^§^
No	39,0 (33,8-44,4)		24,2 (19,8-29,1)		16,1 (12,5-20,5)	
Sí	48,0 (44,5-51,6)		26,2 (23,2-29,4)		17,7 (15,2-20,6)	
**Conductuales**						
**Consumen drogas ilícitas (N=1077)**		<0,001^‡^		<0,001^‡^		0,002^‡^
No	43,6 (40,6-46,7)		24,1 (21,5-26,8)		16,3 (14,1-18,7)	
Sí	70,3 (58,1-80,2)		46,9 (35,1-59,1)		31,3 (21,1-43,6)	
**Fuman (N=1079)**		<0,001^§^		0,016^§^		0,004^§^
No	41,2 (37,9-44,6)		23,4 (20,6-26,4)		15,2 (12,9-17,8)	
Sí	60,7 (53,3-67,6)		32,6 (26,1-39,8)		24,2 (18,4-31,0)	
Ex-fumadores	51,9 (41,0-62,7)		31,7 (22,4-42,7)		24,1 (15,9-34,7)	
**Consumen alcohol (N=1086)**		<0,001^‡^		0,005^‡^		0,098^§^
No	38,6 (34,6-42,8)		21,8 (18,5-25,5)		15,3 (12,5-18,6)	
Sí	51,6 (47,5-55,8)		29,3 (25,6-33,2)		19,1 (16,0-22,6)	
**Parejas celosas (N=1086)**		<0,001^‡^		<0,001^‡^		<0,001^‡^
No	40,3 (37,0-43,8)		21,6 (18,9-24,6)		14,2 (11,9-16,8)	
Sí	58,6 (52,9-64,2)		36,6 (31,2-42,3)		25,5 (20,8-30,9)	
**Ya sintieron miedo de sus parejas (N=1086)**		<0,001^‡^		<0,001^‡^		<0,001^‡^
No	41,5 (38,5-44,7)		20,9 (18,4-23,5)		14,2 (12,1-16,5)	
Sí	77,0 (68,3-83,8)		66,4 (57,2-74,4)		43,4 (34,5-52,6)	
**Parejas controladoras (N=1086)**		<0,001^‡^		<0,001^‡^		<0,001^‡^
No	38,7 (35,4-42,0)		21,0 (18,4-23,9)		13,7 (11,5-16,2)	
Sí	66,3 (60,3-71,8)		40,3 (34,5-46,4)		28,7 (23,5-34,5)	
**Se rehúsan a usar preservativos**		<0,001^‡^		<0,001^‡^		<0,001^‡^
No	41,6 (38,5-44,9)		23,0 (20,4-25,9)		13,5 (11,4-15,9)	
Sí	62,8 (55,6-69,5)		38,3 (31,5-45,5)		35,5 (28,9-42,7)	

*P = Prevalencia; ^†^IC95% = Intervalo de confianza del 95%; ^‡^Prueba exacta de Fisher; ^§^Prueba de chi-cuadrado

Después de los ajustes, en la Tabla 3 se indica que las mujeres con parejas de más edad (al menos 41 años) presentaron una prevalencia de violencia psicológica 19% más baja. Además, este tipo de abuso fue más frecuente entre mujeres con parejas pertenecientes al grupo étnico/color de piel negro (RP: 1,24; IC95%: 1,06-1,44) y que no tenían empleo (RP: 1,25; IC95%: 1,08-1,45). Las parejas fumadoras (RP: 1,17; IC95%: 1,01-1,36), ex-fumadoras (RP: 1,30; IC95%: 1,04-1,61) y que consumían alcohol (RP: 1,19; IC95%: 1,04-1,36) fueron representativas de las características que demuestran mayor prevalencia de violencia psicológica en comparación con el grupo de referencia. Las mujeres que habían sentido miedo de sus parejas (RP: 1,42; IC95%: 1,24-1,64) y con parejas controladoras (RP: 1,48; IC95%: 1,29-1,70) y parejas que se rehusaban a usar preservativos durante las relaciones sexuales (RP: 1,24; IC95%: 1,08-1.42) se encontraron entre las que habían sufrido más violencia psicológica, factor condicionado por posibles factores de confusión (p<0,05).

**Tabla 3:** Análisis ajustado y no ajustado correspondiente a la prevalencia de violencia psicológica a lo largo de la vida de acuerdo con las características de las parejas. Vitória, ES, Brasil, 2022

Características	Análisis no ajustado		Análisis ajustado	
	RP* (IC95%^†^)	Valor p	RP* (IC95%^†^)	Valor p
**Grupo etario**		<0,001		0,002
Hasta 40 años	Ref.^‡^		Ref.^‡^	
Al menos 41 años	0,79 (0,69-0,89)		0,81 (0,71-0,93)	
**Grupo étnico/Color de piel**		0,001		0,029
Blanco	Ref.^‡^		Ref.^‡^	
Negro	1,37 (1,16-1,60)		1,24 (1,06-1,44)	
Mestizo	1,15 (0,99-1,34)		1,09 (0,94-1,26)	
**Años de estudio**		0,033		0,697
Hasta 8	1,18 (1,01-1,37)		0,97 (0,82-1,14)	
Al menos 9	Ref.^‡^		Ref.^‡^	
**Ingresos familiares per cápita (en terciles)**		<0,001		0,232
1^er^ tercil - El nivel más pobre	1,37 (1,16-1,61)		1,15 (0,97-1,36)	
2^o^ tercil	1,10 (0,92-1,32)		1,04 (0,88-1,23)	
3^er^ tercil - El nivel más rico	Ref.^‡^		Ref.^‡^	
**Tienen empleo**		0,009		0,004
No	0,81 (0,70-0,95)		1,25 (1,08-1,45)	
Sí	Ref.^‡^		Ref.^‡^	
**Consumen drogas ilícitas**		<0,001		0,854
No	Ref.^‡^		Ref.^‡^	
Sí	1,61 (1,35-1,92)		1,02 (0,82-1,27)	
**Fuman**		<0,001		0,016
No	Ref.^‡^		Ref.^‡^	
Sí	1,47 (1,27-1,70)		1,17 (1,01-1,36)	
Ex-fumadores	1,26 (1,00-1,58)		1,30 (1,04-1,61)	
**Consumen alcohol**		<0,001		0,012
No	Ref.^‡^		Ref.^‡^	
Sí	1,34 (1,17-1,53)		1,19 (1,04-1,36)	
**Parejas celosas**		<0,001		0,565
No	Ref.^‡^		Ref.^‡^	
Sí	1,45 (1,28-1,65)		1,04 (0,90-1,22)	
**Ya sintieron miedo de sus parejas**		<0,001		<0,001
No	Ref.^‡^		Ref.^‡^	
Sí	1,85 (1,64-2,10)		1,42 (1,24-1,64)	
**Parejas controladoras**		<0,001		<0,001
No	Ref.^‡^		Ref.^‡^	
Sí	1,71 (1,52-1,94)		1,48 (1,29-1,70)	
**Se rehúsan a usar preservativos**		<0,001		0,002
No	Ref.^‡^		Ref.^‡^	
Sí	1,51 (1,32-1,72)		1,24 (1,08-1,42)	

*RP = Razón de prevalencia; ^†^IC95% = Intervalo de confianza del 95%; ^‡^Ref. = Grupo de referencia

En la Tabla 4, los resultados del análisis ajustado demuestran que las mujeres con parejas de más edad (al menos 41 años) presentaron una prevalencia de violencia física a lo largo de la vida un 19% más baja que aquellas con parejas más jóvenes (p<0,05). Las mujeres con parejas de raza negra sufrieron un 33% más violencia física; además, la prevalencia de esa condición fue un 73% más alta entre las que tenían parejas más pobres, es decir, hombres incluidos en el primer tercil en cuanto a ingresos familiares per cápita. Se descubrió que la prevalencia de sufrir violencia física a lo largo de la vida entre las mujeres con parejas que consumían alcohol fue 1,27 veces más alta que la de las mujeres cuyas parejas no lo hacían. Cabe destacar que las participantes que declararon sentir miedo de sus parejas presentaron mayor prevalencia de violencia física en comparación con aquellas que no sentían ese temor; dicha prevalencia fue 2,52 veces más alta. La prevalencia de violencia física a lo largo de la vida fue un 30% más elevada entre las mujeres que tenían parejas controladoras en comparación con las participantes cuyas parejas no lo eran (p<0,05).

**Tabla 4:** Análisis ajustado y no ajustado correspondiente a la prevalencia de violencia física a lo largo de la vida de acuerdo con las características de las parejas. Vitória, ES, Brasil, 2022

Características	Análisis no ajustado		Análisis ajustado	
	RP* (IC95%^†^)	Valor p	RP* (IC95%^†^)	Valor p
**Grupo etario**		0,040		0,037
Hasta 40 años	Ref.^‡^		Ref.^‡^	
Al menos 41 años	0,81 (0,66-0,99)		0,81 (0,67-0,99)	
**Grupo étnico/Color de piel**		<0,001		0,085
Blanco	Ref.^‡^		Ref.^‡^	
Negro	1,73 (1,33-2,24)		1,33 (1,02-1,74)	
Mestizo	1,59 (1,25-2,02)		1,25 (0,98-1,59)	
**Años de estudio**		<0,001		0,590
Hasta 8	1,59 (1,28-1,97)		1,06 (0,85-1,33)	
Al menos 9	Ref.^‡^		Ref.^‡^	
**Ingresos familiares per c*á*pita (en terciles)**		<0,001		<0,001
1^er^ tercil - El nivel más pobre	2,05 (1,58-2,66)		1,73 (1,34-2,24)	
2^o^ tercil	1,17 (0,87-1,58)		1,12 (0,84-1,50)	
3^er^ tercil - El nivel más rico	Ref.^‡^		Ref.^‡^	
**Tienen empleo**		0,484		
No	0,92 (0,73-1,16)		-	-
Sí	Ref.^‡^		-	-
**Consumen drogas ilícitas**		<0,001		0,323
No	Ref.^‡^		Ref.^‡^	
Sí	1,95 (1,47-2,58)		1,18 (0,85-1,63)	
**Fuman**		0,013		0,072
No	Ref.^‡^		Ref.^‡^	
Sí	1,40 (1,09-1,78)		0,94 (0,74-1,21)	
Ex-fumadores	1,35 (0,96-1,92)		1,43 (1,03-2,00)	
**Consumen alcohol**		0,005		0,017
No	Ref.^‡^		Ref.^‡^	
Sí	1,34 (1,09-1,65)		1,27 (1,04-1,54)	
**Parejas celosas**		<0,001		0,620
No	Ref.^‡^		Ref.^‡^	
Sí	1,69 (1,38-2,07)		1,06 (0,84-1,33)	
**Ya sintieron miedo de sus parejas**		<0,001		<0,001
No	Ref.^‡^		Ref.^‡^	
Sí	3,18 (2,66-3,81)		2,52 (2,02-3,13)	
**Parejas controladoras**		<0,001		0,019
No	Ref.^‡^		Ref.^‡^	
Sí	1,92 (1,57-2,34)		1,30 (1,04-1,62)	
**Se rehúsan a usar preservativos**		<0,001		0,079
No	Ref.^‡^		Ref.^‡^	
Sí	1,66 (1,33-2,07)		1,22 (0,98-1,52)	

*RP = Razón de prevalencia; ^†^IC95% = Intervalo de confianza del 95%; ^‡^Ref. = Grupo de referencia

En la Tabla 5 se indica que las mujeres con parejas de raza negra y mestiza presentaron valores de prevalencia 75% y 69% más altos que las mujeres cuyas parejas eran de piel blanca. Además, las participantes que declararon sentir miedo de sus parejas (RP: 1,97; IC95%: 1,48-2,63) y que consideraban que eran personas controladoras (RP: 1,43; IC95%: 1,09-1,86) también presentaron mayor prevalencia de dicho abuso. Rehusarse a utilizar preservativos estuvo asociado a una incidencia 2,18 veces más alta de violencia sexual perpetrada por las parejas sexuales (p<0,05).

**Tabla 5:** Análisis ajustado y no ajustado correspondiente a la prevalencia de violencia sexual a lo largo de la vida de acuerdo con las características de las parejas. Vitória, ES, Brasil, 2022

Características	Análisis no ajustado			Análisis ajustado	
	RP* (IC95%^†^)	Valor p	RP* (IC95%^†^)	Valor p
**Grupo etario**		0,116		0,190
Hasta 40 años	Ref.^‡^		Ref.^‡^	
Al menos 41 años	0,81 (0,62-1,05)		0,84 (0,64-1,09)	
**Grupo étnico/Color de piel**		<0,001		0,001
Blanco	Ref.^‡^		Ref.^‡^	
Negro	1,99 (1,41-2,79)		1,75 (1,26-2,43)	
Mestizo	1,82 (1,33-2,49)		1,69 (1,25-2,29)	
**Años de estudio**		0,004		0,750
Hasta 8	1,52 (1,14-2,01)		1,05 (0,77-1,43)	
Al menos 9	Ref.^‡^		Ref.^‡^	
**Ingresos familiares per c*á*pita (en terciles)**		0,001		0,555
1^er^ tercil - El nivel más pobre	1,81 (1,30-2,53)		1,20 (0,85-1,72)	
2^o^ tercil	1,27 (0,88-1,82)		1,08 (0,75-1,54)	
3^er^ tercil - El nivel más rico	Ref.^‡^		Ref.^‡^	
**Tienen empleo**		0,519		
No	0,91 (0,68-1,22)		-	-
Sí	Ref.^‡^		-	-
**Consumen drogas ilícitas**		0,001		0,421
No	Ref.^‡^		Ref.^‡^	
Sí	1,92 (1,30-2,83)		1,20 (0,77-1,86)	
**Fuman**		0,004		0,171
No	Ref.^‡^		Ref.^‡^	
Sí	1,59 (1,17-2,16)		1,18 (0,87-1,59)	
Ex-fumadores	1,58 (1,04-2,42)		1,47 (0,95-2,25)	
**Consumen alcohol**		0,099		0,493
No	Ref.^‡^		Ref.^‡^	
Sí	1,25 (0,96-1,62)		1,10 (0,84-1,44)	
**Parejas celosas**		<0,001		0,804
No	Ref.^‡^		Ref.^‡^	
Sí	1,80 (1,39-2,33)		1,04 (0,78-1,38)	
**Ya sintieron miedo de sus parejas**		<0,001		<0,001
No	Ref.^‡^		Ref.^‡^	
Sí	3,06 (2,35-3,97)		1,97 (1,48-2,63)	
**Parejas controladoras**		<0,001		0,009
No	Ref.^‡^		Ref.^‡^	
Sí	2,10 (1,62-2,72)		1,43 (1,09-1,86)	
**Se rehúsan a usar preservativos**		<0,001		<0,001
No	Ref.^‡^		Ref.^‡^	
Sí	2,63 (2,04-3,40)		2,18 (1,68-2,83)	

*RP = Razón de prevalencia; ^†^IC95% = Intervalo de confianza del 95%; ^‡^Ref. = Grupo de referencia

## Discusión

En el estudio se demostró que existe una asociación entre las características sociodemográficas y conductuales de las parejas sexuales de las mujeres y la incidencia de violencia contra ellas a lo largo de la vida. Se descubrió que las mujeres con parejas que tenían conductas controladoras, eran celosas, se rehusaban a utilizar preservativos durante las relaciones sexuales, fumaban o eran ex-fumadores, consumían alcohol, no tenían empleo y pertenecían al grupo étnico/color de piel negro y al primer tercil en cuanto a ingresos familiares per cápita (el nivel más pobre) presentaron mayor prevalencia de violencia (psicológica, física y/o sexual) a lo largo de la vida. Los hallazgos se condicen con estudios nacionales desarrollados con usuarias de servicios de atención primaria^3^ y puérperas en una maternidad para embarazos de riesgo habitual^16^.

Después de los ajustes, las mujeres con parejas más jóvenes (hasta 40 años) presentaron mayor prevalencia de violencia psicológica y física. Los resultados de este estudio corroboran un trabajo nacional desarrollado en el año 2003 con 251 usuarias de servicios de salud en Porto Alegre (Rio Grande do Sul), en el que se demostró que existe una asociación entre la incidencia de violencia psicológica y física y parejas de menos de 30 años de edad^17^. En un estudio internacional se indicó que las mujeres con parejas de más edad (al menos 60 años) presentaron 43% más probabilidades de sufrir violencia de pareja que las que tenían parejas más jóvenes (18-29 años)^4^.

La prevalencia más alta de violencia física se observó entre las mujeres con parejas incluidas en el 1^er^tercil de ingresos, en comparación con quienes pertenecían al tercil más rico. Resulta crucial contextualizar que la asociación entre mayor nivel de pobreza y mayor incidencia de violencia contra las mujeres no debe interpretarse como una relación causal directa sino como un reflejo de condiciones estructurales. A menudo, los contextos signados por la pobreza implican mayor estrés financiero, condiciones precarias de vivienda y menos acceso a redes de apoyo y servicios de protección, factores que pueden intensificar conflictos y reducir la cantidad de alternativas para resoluciones no violentas. Además, la violencia perpetrada por hombres pobres tiende a ser más visible y menos tolerada por la sociedad, mientras que es posible que conductas similares en grupos socioeconómicos más altos se consideren normales o se oculten, afectando así el reconocimiento y la denuncia de casos de violencia^18^.

Otra de las características de los atacantes que estuvo asociada a mayores probabilidades de cometer actos de violencia psicológica, física y sexual fue pertenecer al grupo étnico/color de piel negro. La realidad socioeconómica y racial de Brasil indica que los hombres brasileños de raza negra tienen niveles de estudio más bajos y empleos menos prestigiosos que sus pares de raza blanca^19^. Los hombres y las mujeres con menos estudios están más expuestos a sufrir violencia: el 91,8% de las víctimas de asesinato son hombres y el 75,7% son hombres de raza negra^20^.

Es preciso considerar que este resultado puede reflejar sesgo racial en la percepción de conductas violentas, donde los estereotipos asocian a hombres de raza negra con mayor agresividad, derivando en que la violencia se reconozca con más rapidez o intensidad cuando el agresor es de raza negra que cuando su piel es de color blanco, incluso en situaciones similares. Este fenómeno ya se ha observado en estudios sobre la forma en la que la raza influencia la determinación de casos de violencia y la culpabilidad en violencia de pareja; por ejemplo, los trabajos de investigación sobre cómo se percibe la violencia de pareja y las decisiones de jurados en escenarios diferenciados racialmente indican que la raza de los agresores altera la forma en la que se evalúa la violencia^21^.

Además, las mujeres con parejas que no trabajaban presentaron mayor prevalencia de violencia psicológica a lo largo de la vida. Los hombres sin empleo estable tienden a ejercer mayor control coercitivo sobre sus parejas, considerando un contexto en el que el estrés económico puede ser un predictor significativo de violencia^5^.

Se hizo evidente que las mujeres que autodeclararon sentir miedo de sus parejas presentaron mayor prevalencia de violencia (psicológica, física y sexual) a lo largo de la vida. A menudo, las relaciones interpersonales violentas están signadas por temor y conductas controladoras entre los integrantes de una pareja^1^; por ese motivo, el hecho de que un hombre presente conductas violentas capaces de justificar el miedo que sienten estas mujeres es un factor relevante para sufrir consecuencias físicas y psicológicas, tanto antes como después de materializarse la violencia. Dichas conductas opresivas por parte de los hombres están relacionadas con la cultura patriarcal y su estructura, en la que los hombres y las mujeres son inseparables, reproduciendo así el rol de las mujeres subordinadas a los hombres^22^.

Se deben considerar los diversos aspectos que pueden desencadenar miedo en las mujeres ante situaciones de violencia doméstica; además, el temor surge como un elemento inmovilizador e incapacita cualquier reacción para poder interrumpir el ciclo^8^. Como ya se analizó, el temor también puede ser resultado de una prolongada serie de amenazas del agresor y también infligir posibles daños a corto y largo plazo, siendo mayor debido a la sensación de impunidad con la que el Estado responde a la violencia^8^. También puede basarse en amenazas a la propia vida o a la de personas cercanas, como hijos y familiares^22^.

Con respecto al uso de preservativos, en el estudio se descubrió que las mujeres con parejas que se rehusaban a utilizarlos presentaron mayor prevalencia de violencia psicológica y sexual. En consonancia con los hallazgos de este estudio, la literatura reafirma la evidencia indicada^3^ ^,^ ^16^ ^-^ ^17^. El resultado después de los ajustes que se encontró en este estudio para la violencia sexual fue idéntico al descubierto en otro trabajo realizado con usuarias de Atención Primaria de la Salud en la misma ciudad^3^. En el estudio se demostró que las mujeres con parejas que se rehusaban a utilizar preservativos tuvieron nueve veces más probabilidades de sufrir violencia sexual, incluso durante sus embarazos^16^. La dificultad para negociar el uso de preservativos y/o de otros métodos anticonceptivos señala la fragilidad del poder de decisión de las mujeres sobre su propia salud sexual y reproductiva, lo que pone de manifiesto un contexto en el que este poder reside en los hombres^3^ ^,^ ^16^ ^,^ ^23^.

Además del rol del dominio masculino en cuanto a la decisión de utilizar preservativos, también se hace evidente su dominación sobre la salud sexual de las mujeres. Se sabe que los hombres que entienden que utilizar preservativos previene enfermedades como el VIH también tienen una influencia más positiva sobre sus relaciones sexuales con mujeres, en oposición a los que no conocen métodos de protección sexual^24^. Además, en un estudio realizado en Haití se demostró que el hecho de que las mujeres se rehusaran a tener relaciones sexuales estuvo asociado a violencia física y a índices más bajos en cuanto a uso de preservativos de forma concomitante^24^.

Con respecto a las conductas controladoras masculinas, las mujeres que viven en medio de una relación violenta pueden experimentar distanciamiento social de sus familiares y amigos, puesto que están constantemente sometidas a vigilancia e imposición de reglas que infringen los derechos humanos y perjudican su calidad de vida. Además del control social, las mujeres tienen poder limitado en circunstancias sexuales; eso dificulta crear un espacio para negociar el uso de preservativos y las pone en riesgo de violencia^1^ ^,^ ^23^.

Además de las desigualdades de género y en materia de relaciones de poder dentro de la estructura social y de relaciones, la desigualdad social también es un factor importante en la no interrupción del ciclo de violencia. Factores como dependencia financiera^25^, nivel educativo bajo^23^, preocupación acerca del futuro de los hijos y dependencia emocional son determinantes para que las mujeres permanezcan en el ciclo de violencia^8^ ^,^ ^26^. Otro factor relevante en cuanto a la vulnerabilidad de permanecer en el contexto de violencia está relacionado con haber experimentado otros tipos de violencia doméstica en la vida, al igual que un contexto familiar violento que puede influenciar la naturalización de la violencia^27^.

En este estudio se detectó mayor prevalencia de violencia psicológica contra las mujeres perpetrada por hombres que eran fumadores o ex-fumadores, resultado que también se observó en un estudio realizado en la ciudad de Shenzhen (China)^28^. Probablemente, esto se deba a factores asociados al hábito de fumar, como estrés, impulsividad y consumo simultáneo de alcohol y otras sustancias, que son ampliamente reconocidos como factores de riesgo para la violencia^28^ ^-^ ^29^.

En este estudio, el hecho de que las parejas de las mujeres consumieran alcohol estuvo asociado a mayor prevalencia de violencia psicológica y física. El alcohol es una sustancia psicoactiva, y su consumo afecta conexiones y vías neuronales. Además, sus efectos pueden verse intensificados dependiendo de la cantidad consumida, con posibles consecuencias que derivan en lo siguiente: déficits de atención, cognición y destreza; mayor impulsividad; perfil de personalidad agresivo; pérdida del control conductual; y exacerbación de reacciones^30^. Por lo tanto, consumir alcohol puede aumentar el riesgo de violencia debido a la posibilidad de conflictos y externalización de conductas violentas, expresando el aspecto impulsivo y agresivo de parejas sexuales que poseen una construcción social en la que el machismo y el patriarcado están enraizados^3^ ^,^ ^5^ ^,^ ^16^.

En Brasil, los hombres consumen alcohol más excesivamente que las mujeres^30^. El riesgo atribuible y los costos hospitalarios relacionados con la violencia son elevados; además, el costo promedio total de la violencia interpersonal entre los años 2010 y 2018 debido al alcohol puede estimarse en más de 17 millones de dólares^26^. Se entiende que el fenómeno en estudio es multidimensional. Sin embargo, Brasil es uno de los países que no ha avanzado dinámicamente en materia de implementar políticas públicas para abordar el consumo nocivo de alcohol, sin ningún contacto intersectorial destinado a reducir el riesgo de violencia^31^.

La incidencia de violencia a lo largo de la vida puede causar varias consecuencias físicas, psicológicas y sociales a corto y largo plazo en el caso de mujeres con antecedentes; además, combinado con dependencia financiera y emocional, el miedo puede obstaculizar el proceso necesario para interrumpir el ciclo de violencia^1^. En consecuencia, los profesionales de la salud representan un contacto de comunicación crítico para estas personas^32^. Sin embargo, para poder proporcionar atención efectiva y eficiente a mujeres en situaciones de violencia, es esencial que estos profesionales desarrollen su punto de vista sobre este grave problema de salud pública, que abarca distintas dimensiones de la vida humana (social, espiritual, física, mental y biológica). También es necesario que esta perspectiva profesional sea de naturaleza interprofesional e intersectorial para que se implementen las principales derivaciones de acuerdo con las necesidades reales de las mujeres atendidas^33^. También es importante destacar las notificaciones obligatorias que deben realizar estos profesionales ante casos o sospechas de violencia. Estas notificaciones se han convertido en una herramienta clave para la vigilancia epidemiológica y para formular políticas públicas de prevención e intervención^34^.

Los resultados de este estudio revisten gran relevancia para los profesionales dedicados a la Enfermería clínica y a otras áreas de la salud, especialmente en la atención de mujeres durante consultas ginecológicas y obstétricas, ya que refuerzan la importancia de conocer los perfiles de las parejas sexuales, considerando que ciertas características pueden derivar en mayor riesgo de violencia. En consecuencia, los hallazgos refuerzan la necesidad de diseñar procedimientos de recolección de datos más completos y holísticos, al igual que la de informar este problema e incluir a las mujeres en redes de atención y protección.

Este estudio es el primer trabajo poblacional que describe el perfil de las parejas sexuales actuales entre mujeres que viven en una capital de la región sudeste del país. La violencia se midió con un instrumento validado y se garantizó privacidad a las mujeres para que respondieran la totalidad del cuestionario. El estudio aporta a la literatura nacional e internacional sobre el perfil de parejas de mujeres con mayor prevalencia de violencia a lo largo de la vida (literatura que cuenta con varios vacíos); por lo tanto, la información presentada puede asistir en la planificación e implementación de políticas públicas intersectoriales para combatir la violencia contra las mujeres.

Es necesario mencionar algunas limitaciones, como causalidad inversa, puesto que se trata de un estudio transversal y la temporalidad de la relación está comprometida, motivo por el cual resulta muy importante realizar estudios longitudinales. El sesgo de deseabilidad social puede hacer que las mujeres que son víctimas de violencia doméstica adapten sus declaraciones para cumplir con las normas sociales o con las expectativas de los investigadores. Eso puede terminar minimizando la gravedad de la violencia al omitir episodios o describir al perpetrador desde una perspectiva menos negativa, especialmente cuando las mujeres sienten temor de ser juzgadas o estigmatizadas. Además, los sentimientos de culpa y dependencia emocional o económica pueden influenciar respuestas más defensivas con respecto al perpetrador. Por lo tanto, este sesgo puede poner en riesgo la exactitud y completitud de los datos sobre la violencia sufrida. También puede haber confusión residual por los motivos ya mencionados.

## Conclusión

Los resultados demostraron que existe una asociación entre la violencia contra las mujeres y las características socioeconómicas y conductuales de sus parejas sexuales. Se sabe que el fenómeno de la violencia es complejo y multicausal; sin embargo, este estudio demuestra que ciertas características (especialmente las conductuales) de las parejas sexuales tienen una asociación más intensa con la incidencia de la violencia. Estos datos presentan un camino a seguir para implementar medidas de intervención basadas en evidencia, ya que las conductas pueden modificarse si se aplican intervenciones eficientes. Los hallazgos destacan la importancia de implementar acciones intersectoriales para promover una cultura de paz y abordar los temas de la violencia y la desigualdad social y de género, además de la necesidad de reducir el consumo de alcohol, tabaco y otras drogas.

## Data Availability

Los conjuntos de datos relacionados con este artículo estarán disponibles previa solicitud al autor correspondiente.
